# Quantifying extreme failure scenarios in transportation systems with graph learning

**DOI:** 10.1016/j.patter.2025.101209

**Published:** 2025-03-14

**Authors:** Mingxue Guo, Tingting Zhao, Jianxi Gao, Xin Meng, Ziyou Gao

**Affiliations:** 1 School of Systems Science, Beijing Jiaotong University, Beijing 100044, China; 2 Department of Computer Science, Rensselaer Polytechnic Institute, Troy, NY 12180, USA

**Keywords:** extreme failure, importance sampling, graph autoencoder, sampling efficiency, road network

## Abstract

Statistical analysis of extreme events in complex engineering systems is essential for system design and reliability and resilience assessment. Due to the rarity of extreme events and the computational burden of system performance evaluation, estimating the probability of extreme failures is prohibitively expensive. Traditional methods, such as importance sampling, struggle with the high cost of deriving importance sampling densities for numerous components in large-scale systems. Here, we propose a graph learning approach, called importance sampling based on graph autoencoder (GAE-IS), to integrate a modified graph autoencoder model, termed a criticality assessor, with the cross-entropy-based importance sampling method. GAE-IS effectively decouples the criticality of components from their vulnerability to disastrous events in the workflow, demonstrating notable transferability and leading to significantly reduced computational costs of importance sampling in large-scale networks. The proposed methodology improves sampling efficiency by one to two orders of magnitude across several road networks and provides more accurate probability estimations.

## Highlights

• Proposes a sampling framework that decouples component criticality from failure risk
• Introduces a transferable component criticality assessor
• Enhances sampling efficiency for extreme events in large-scale transport systems
• Identifies consistent feature patterns influencing link criticality across networks

## The bigger picture

Extreme events, like extreme weather, earthquakes, and other disasters, although rare, can have catastrophic effects on critical infrastructure such as transportation networks and power grids. Predicting the impact of these “black swan” events and estimating the probability of extreme failures has long been a challenge due to the high computational cost of traditional methods. Here, the researchers introduce an approach that leverages graph learning techniques to reduce computational burden and improve the accuracy of extreme failure probability estimation. This method could be applied to evaluate the resilience of diverse urban infrastructures, including power, transportation, and water supply systems.

## Teaser

Analyzing extreme events in large-scale engineering systems is computationally expensive due to the rarity of such events. To overcome this, the authors propose the GAE-IS method, which integrates a modified graph autoencoder with cross-entropy-based importance sampling. Their approach significantly enhances sampling efficiency and accuracy in extreme scenario probability estimation, with experiments on road networks from four cities demonstrating improvements by one to two orders of magnitude.

## Introduction

The vulnerability of engineered systems to extreme events poses significant risks, including substantial loss of life, economic damage, and threats to national security.^1^^,^^2^ These extreme events range from catastrophic disasters that can devastate infrastructure such as power grids and transportation networks, leading to the failure of critical lifelines,^3^^,^^4^ to accidents caused by aging components or system malfunctions^5^^,^^6^ and severe cascading failures triggered by load fluctuations.^7^^,^^8^ Although these extreme events have a low probability of occurrence, residing in the tail of the probability distribution, their social and economic impacts are often immense and unacceptable. Therefore, assessing the reliability of engineering systems under such extreme conditions, known as “corner cases,” is essential. This assessment serves as the cornerstone for reliable, robust, and resilient system planning and design,^9^^,^^10^ identifying potential risks and vulnerabilities^11^ and developing effective countermeasures^12^^,^^13^ to mitigate the likelihood or impact of such events. However, given the rarity of extreme events and the challenges in characterizing them,^14^ achieving computational efficiency in reliability or resilience assessments becomes a significant challenge, particularly when using traditional methods such as the Monte Carlo simulation.^15^^,^^16^ For instance, estimating the probability of extreme failure scenarios at a level of $10^{-6}$ with a standard error of 10% would require sample sizes on the order of $10^{8}$. In practice, simulating the performance of complex nonlinear engineering systems under numerous failure scenarios is computationally intensive, presenting significant obstacles to accurately evaluating system reliability and resilience under extreme conditions.

A method that has gained prominence in recent research for rare event analysis is adaptive sampling based on active learning. This approach actively chooses samples by optimizing a predefined acquisition function,^17^ which accelerates the convergence of extreme scenario probability estimates. This method has been applied in the fields of safety and reliability analysis, such as accident rate detection for autonomous vehicles,^18^ reliability assessment of engineering structures,^19^^,^^20^ and the discovery and prediction of rogue ocean waves.^14^ However, this method is data driven and relies on training samples. When evaluating the probability of extreme failures for infrastructure systems under disastrous events, it is necessary to retrain the model whenever there is a change in the type of disaster or in the spatial distribution of the disaster’s intensity.

Another commonly used method is importance sampling,^21^^,^^22^ which has been recognized as an effective method for reducing variance in the statistical analysis of extreme events. This method employs an auxiliary distribution known as the importance sampling density (ISD). By transforming samples of interest, which are unlikely to be obtained from the original distribution, into samples with higher probability density under the ISD, importance sampling significantly improves sampling efficiency related to rare events. This technique has found extensive application in risk and reliability assessments across various domains, such as credit risk evaluation in portfolio investments in the financial sector,^23^^,^^24^ multiscenario driving safety testing for autonomous vehicles,^25^^,^^26^ and vulnerability and reliability assessment for power systems,^27^^,^^28^ as well as other engineered systems characterized by series-parallel structures and complex failure patterns.^29^^,^^30^ These applications highlight the versatility and effectiveness of importance sampling in addressing challenges associated with rare and extreme events.

However, a primary challenge in implementing importance sampling lies in identifying an appropriate ISD, as it significantly affects both the accuracy of probability estimation and the degree of sampling efficiency improvement.^15^^,^^22^ Various methods have been employed to approximate the ISD, including parametric density approximation methods such as Gaussian mixture models^31^^,^^32^ and nonparametric methods like adaptive kernel density estimators.^33^^,^^34^ Among these, the cross-entropy (CE) method is a widely used adaptive sampling technique. It approximates the ISD by minimizing the Kullback-Leibler (KL) divergence between the theoretically optimal ISD and a chosen parametric family of distributions.^35^ Despite its effectiveness, the CE method and similar approaches face limitations when dealing with high-dimensional problems.^36^ As the dimensionality of the variables increases, the number of unknown model parameters and required sample size escalate rapidly.^37^ This poses significant challenges when applying these methods to estimate extreme failure probabilities in large-scale networks with numerous components, where the complexity and computational costs can become prohibitive. For infrastructure systems such as transportation networks, which are often large-scale and complex, resilience assessment and optimization in the existing efforts typically measure resilience using the overall system performance under a specific extreme event^38^ or the mean of system performances under a group of failure scenarios.^39^ Addressing the challenge of evaluating the probability of extreme failures will enable a more comprehensive resilience assessment from the perspective of extreme value statistics.^40^^,^^41^

In this study, we propose a graph learning approach named importance sampling based on graph autoencoder (GAE-IS) to efficiently sample extreme failure scenarios and estimate their probabilities in infrastructure networks, taking transportation networks as an example. The experiments on road networks demonstrate the effectiveness of the proposed methodology, showcasing its ability to offer a computationally feasible approach for assessing the resilience of large-scale infrastructure networks.

### Overview of the GAE-IS approach

**Figure 1:**
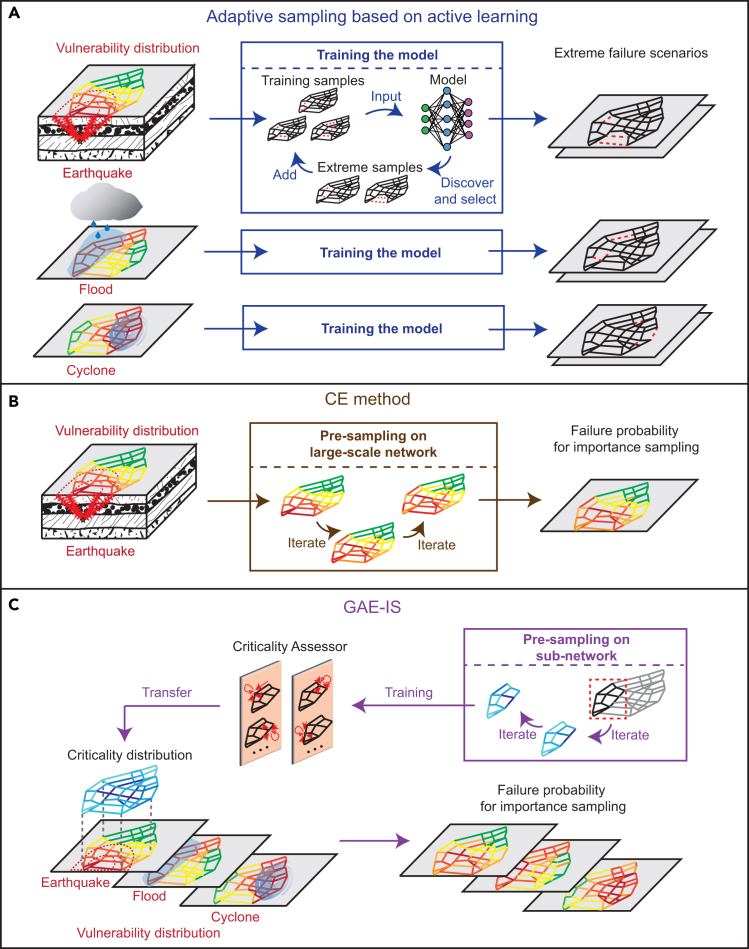
Comparison of GAE-IS (importance sampling based on graph autoencoder) with the existing CE (cross-entropy) method and adaptive sampling method on a road network for estimating the probability of extreme failures under various disaster scenarios

GAE-IS represents an advancement over the traditional CE method and addresses the challenge of sampling difficulties associated with obtaining the ISD function in large-scale networks with numerous components. The proposed GAE-IS method offers two key advantages, as illustrated in Figure 1.

(1) Transferability of the proposed criticality assessor via a graph autoencoder (GAE): we develop a graph learning model based on a GAE, referred to as the criticality assessor, to assess the criticality of network components on overall network functionality. In this study, the criticality of a network component is defined as the extent to which the component’s failure results in the degradation of network performance, taking multiple components’ simultaneous failure into consideration. The criticality assessor, trained on a small-scale sub-network, can be effectively transferred to the large-scale network it belongs to, thereby facilitating the efficient determination of the ISD function without requiring extensive presampling on the larger target network as shown in Figure 1B. Additionally, due to its transferability, the number of parameters in the model remains constant regardless of the scale of the system or the dimensionality of the variables. This effectively addresses the challenges of parameter and sample-size explosion typically faced by existing importance sampling methods, such as the CE method, in high-dimensional systems.
(2) Decoupling criticality from physical failure characteristics in the workflow: we assume that vulnerability is related to the spatial distribution of the disruptive intensity caused by potential hazards. Combined with the structural fragility of components, the resulting risk of component failure is referred to as the vulnerability distribution, as shown in Figure 1A. The criticality of components is independent of the vulnerability distribution. By integrating the spatial distribution of criticality, termed as the criticality distribution, as shown in Figure 1C, with the network’s vulnerability distribution in the context of a catastrophic event, we can derive ISD functions of components for GAE-IS. This approach eliminates the need to retrain the model across various types of disasters or various vulnerability distributions, which are inevitable in the adaptive sampling methods shown in Figure 1A.

GAE-IS is designed to accommodate a range of component failure patterns, including various levels of capacity degradation, and is capable of handling both homogeneous and heterogeneous failure probabilities. Moreover, it takes into account both the network’s topology and its spatial travel demand distribution characteristics. As a result, although focusing on transportation systems in this study, the proposed methodology has the potential to be applied to other infrastructure systems characterized by network topological features and cyber or physical flows transferring in it, such as water distribution, power, and communication systems.

## Methods

### The workflow of GAE-IS

Road transportation networks are represented as directed graphs consisting of nodes and links. Nodes represent intersections and origin-destination (OD) points where travel demand originates or terminates, while links represent road segments. The average travel time (ATT) is used as the system performance indicator to evaluate the post-event system level of service. It measures the extent of functionality loss in the system, thereby aiding in determining whether a failure scenario should be classified as an extreme failure scenario. The ATT is calculated as:

(Equation 1) $$\text{ATT}=\frac{1}{D}\sum_{i}t_{l_{i}}Q_{l_{i}}\text{,}$$

where D represents the total travel demand in the network, $Q_{l_{i}}$ is the traffic volume on link $l_{i}$, and $t_{l_{i}}$ represents the travel time on link $l_{i}$, which is calculated using the Bureau of Public Roads (BPR) function:

(Equation 2) $$t_{l_{i}}=t_{l_{i}0}\times \left(1+{\alpha \times \left(\frac{Q_{l_{i}}}{{CA}_{l_{i}}}\right)}^{\beta }\right)\text{,}$$

where $t_{l_{i}0}$ is the free-flow travel time of link $l_{i}$ and ${CA}_{l_{i}}$ is the link capacity; $t_{l_{i}0}$ and ${CA}_{l_{i}}$ are predetermined by the network configuration given in the transportation network dataset,^42^ and parameters α and β take 0.15 and 4, respectively, referring to the literature.^43^

In our study, $Q_{l_{i}}$ is the output of the traffic assignment model, i.e., user equilibrium (UE)^44^; the formulation of UE can be found in Appendix A in the supplemental methods. UE-based traffic assignment is affected by various factors, including network topology, capacity configurations, spatial distribution of travel demand, and the adaptability of network users to congested traffic conditions. It is a complex nonlinear and self-organizing process, with computational complexity increasing significantly with the size of the network.^45^ This study focuses on failure scenarios with capacity degradations of road segments. Therefore, for each failure scenario, the travel demand is reassigned, given the decreased capacity of affected links to determine the volume $Q_{l_{i}}$ on each link. For the failed links, the parameter ${CA}_{l_{i}}$ in the BPR function is also reduced accordingly. In future work, scenarios with complete link interruption can be studied by reassigning the remaining connected travel demand and imposing a penalty on the disconnected travel demand to calculate the ATT.

Given factors such as geographic location and structural characteristics of road segments, there exists a spatial heterogeneous failure probability τ for each link. We assume that the structural failure of links is mutually independent. Failure scenarios refer to various combinations of failed links within the network, and extreme failure scenarios are defined as those with ATT exceeding the threshold $\theta_{e}$. To set the threshold for extreme failure scenarios, follow these steps. First, sample $5\times 10^{3}$ failure scenarios using a crude Monte Carlo method. Then, fit the right-skewed ATT with a gamma distribution or use kernel density estimators to account for multimodal ATT distributions. The 99.75th and 99.95th percentiles of the ATT distribution are considered as extreme failure thresholds. In practice, the threshold can be adjusted flexibly to meet the specific requirements of the application scenario, and the fitting methods can be further adjusted according to the specific probability distribution characteristics of ATT in the sampled scenarios. Although this provides a reference setting for $\theta_{e}$, fitting the ATT distribution introduces errors, making percentile-based probability estimates inaccurate. Accurate probabilities of extreme failure scenarios still require estimation through the proposed GAE-IS method. Overall, in the context of this study, extreme failure scenarios in the road network are those in which multiple road segments fail simultaneously, resulting in a significant decline in traffic efficiency, i.e., significant increase in ATT.

The criticality of links is unrelated to their structural condition and the spatial distribution of the disruptive intensity. Therefore, we assume that links in the sub-network (training network) have a homogeneous hypothetical failure probability ϵ for the training of the criticality assessor. This allows us to assess the criticality of links and identify those that are critical for network functionality, given the same failure probability among links. By increasing the failure probabilities of these critical links, we have a greater chance of obtaining more extreme failure scenarios. Unlike studies focusing on the impact of individual component failures on system functionality,^46^^,^^47^ our study emphasizes the criticality of links under simultaneous multiple component failures.

**Figure 2:**
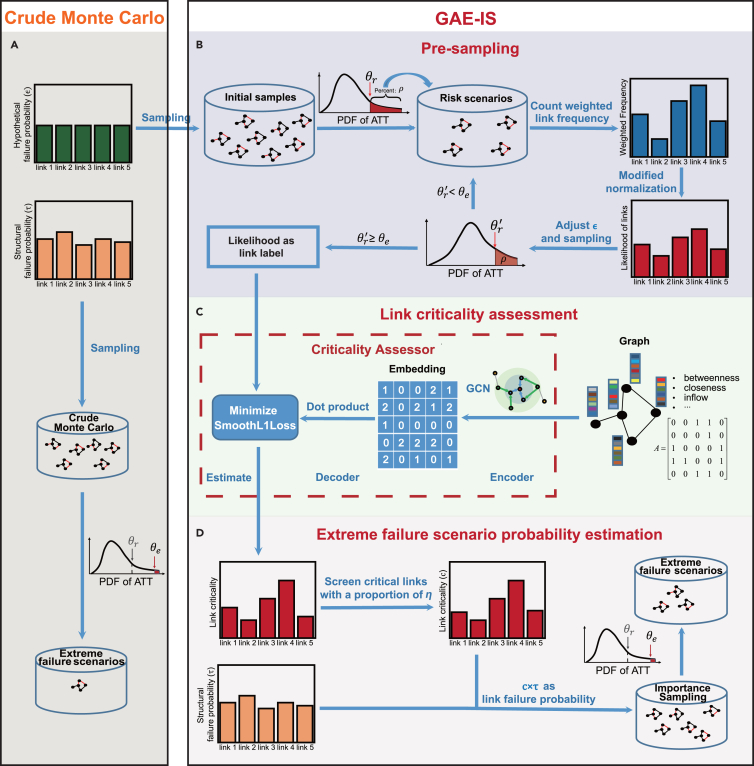
Overview of the GAE-IS workflow This figure illustrates the process for sampling extreme failure scenarios that lead to significant degradation in traffic efficiency due to link failures in a road network. (A) The crude Monte Carlo method used for presampling and serving as a baseline for sampling extreme failure scenarios. (B–D) GAE-IS comprises three key components: (B) presampling on a sub-network using the CE method to estimate the likelihood of each link being part of failed link sets in risk scenarios, (C) assessing link criticality using the criticality assessor, and (D) sampling extreme failure scenarios based on the link criticality provided by the criticality assessor.

The crude Monte Carlo is employed to randomly sample $N_{t}$ network failure scenarios with a given link failure probability, ϵ, in the training network, creating the initial sample set (Figure 2A). A predetermined percentage (i.e., the ρ percentage) of these samples, which show substantial degradation in network performance, is selected as risk scenarios. The threshold for ATT corresponding to these risk scenarios is denoted as $\theta_{r}$. The likelihood of each link appearing in the set of failed links within these risk scenarios is calculated, where a higher likelihood indicates a more critical link. This likelihood is utilized to adjust the hypothetical failure probability of the link to increase it for more critical links and decrease it for less critical links. Then, another set of $N_{t}$ samples is generated, and the risk scenarios are updated with a new risk scenario threshold, $\theta_{r}^{\prime }$. This iterative process continues until the risk scenario threshold $\theta_{r}^{\prime }$, associated with the ρ percentage of the samples, exceeds $\theta_{e}$ (for details of the iterative process refer to Appendix B in supplemental methods). This iterative procedure is referred to as presampling, as shown in Figure 2B.

Next, the criticality assessor uses inputs such as the road network’s adjacency matrix and node attributes related to multiple topological centrality metrics and traffic flow (details in the next sub-section) to learn the feature representation of each link’s impact on network performance degradation, as shown in Figure 2C. It estimates and outputs the likelihood of each link being part of the set of failed links in risk scenarios, thereby quantifying the criticality of the link. The estimated criticality is then used as a coefficient to adjust links’ structural failure probabilities, as shown in Figure 2D. Links are sorted by their criticality in descending order. The top η proportion of links is considered critical, while the bottom η proportion is deemed noncritical, leading to adjustments in their failure probabilities. For details on how to modify the estimated criticality based on parameter η, please refer to Appendix B in the supplemental methods. This approach integrates link criticality with failure risk to derive the ISD function for each link, which is associated with the adjusted failure probability of that link. Ultimately, the ISD function for failure scenarios is determined by the ISD function of each link. When the structural failure probability of a link changes, there is no need to reassess the criticality. Thus, the GAE-IS can be applied to networks considering various types of disasters and spatial vulnerability distributions, enhancing computational efficiency in assessing system resilience across different vulnerability characteristics.

It’s worth mentioning that transferring a trained criticality assessor to another network requires providing only the adjacency matrix and node attributes of that network to obtain the new criticality of links, without the need of presampling.

### The criticality assessor for obtaining the ISD function

The criticality assessor is designed to assess link criticality and hence to obtain the ISD function for sampling extreme failure scenarios. This model is based on the GAE framework, an unsupervised deep learning model proposed by Kipf and Welling.^48^ The GAE entails utilizing the known adjacency matrix of an incomplete graph and the feature matrix of nodes as inputs, applying graph convolution to encode and learn node representations and then reconstructing the original network using a decoder. The model parameters are optimized by minimizing the graph reconstruction error to address link prediction tasks.

In the proposed criticality assessor, the node feature matrix consists of both topological metrics and traffic flow attributes. Table 1 provides a detailed description of these features. Each node is represented by a 12-dimensional feature vector. During the data preprocessing stage, each feature dimension of the node is standardized. To encode the input data, we use two layers of graph convolutional networks followed by two fully connected layers, with link capacity serving as the edge attribute. Rather than tackling link prediction tasks with a traditional GAE model, the proposed criticality assessor employs the inner product of node embedding vectors in its decoding module to estimate the likelihood of link occurrence within a set of failed links in risk scenarios.

**Table 1:** Node features of the road network

Feature category	Feature	Description	Feature function
Topological centrality metrics	in-degree	number of incoming edges	reflects a node’s connectivity
	out-degree	number of outgoing edges	
	betweenness centrality	number of shortest paths through a node	reflects the node’s role in efficiently connecting nodes
	closeness centrality	the mean distance from the node to all other nodes in the network	reflects the position of a node in the network
	eigenvector centrality	determined by the centrality of the node’s neighbors	reflects the importance of a node’s neighbors
Traffic flow attributes	inflow	flow into the node	reflects the node’s load
	outflow	flow out of the node	
	a binary indicator for whether the node serves as an origin or a destination point for travel demands	1 for yes, 0 for no	reflects the role of the node in travel demand distribution
	the remaining capacity of a node as a starting point	difference between the sum of traffic capacity of all links starting at the node and the sum of traffic flow on links starting at the node	reflects the redundancy in capacity of roads connected to the node
	the remaining capacity of a node as an ending point	difference between the sum of traffic capacity of all links ending at the node and the sum of traffic flow on links ending at the node	
	the free-flow speed of a node as a starting point	sum of the free-flow speeds of all links starting at the node	reflects the hierarchy of roads connected to the node; higher-hierarchy roads are typically designed to have higher free-flow speeds
	The free-flow speed of a node as an ending point	sum of the free-flow speeds of all links ending at the node	

In transportation networks, traffic flow can vary significantly across different directions of the same road segment due to heterogeneous spatial travel demand distributions. Consequently, the impact of a road segment’s failure on network performance may differ depending on its direction. To account for this, this study models the road transportation network as a directed graph. However, the traditional GAE was designed for undirected graphs. For example, using the inner product of endpoint embedding vectors to calculate the probability of a link’s existence does not differentiate between directed links from node i to node j and those from node j to node i. To address this issue, previous studies have developed node embedding methods^49^ or decoding techniques^50^ specifically for directed graphs. We adopt the approach proposed by Ou et al.,^51^ which involves training embedding vectors separately for the starting and ending points of links, thereby allowing us to differentiate bidirectional links.

To train the criticality assessor, we assign a real-valued label to each link in the network. Details of the label generation process are provided in Appendix B in the supplemental methods. The parameter optimization for the criticality assessor is achieved by minimizing the error between the estimated likelihood of links (calculated as the inner product of node embedding vectors) and their corresponding real-valued labels. The specific hyperparameter settings of the model are presented under “experimental setup.”

### Importance sampling theory

Importance sampling is a technique that enhances the number of specific samples by generating samples from an introduced auxiliary distribution.^15^ We consider the objective of estimating the value of $E\left(f\left(x\right)\right)=\int_{D}f\left(x\right)g\left(x\right)dx$, where g(x) is a probability density function defined on $D\subseteq R^{d},g$ is referred to as the nominal distribution, and f represents the integrand. For all x∉D, g(x)=0. If q(x) is a positive probability density function on $R^{d}$, then:

(Equation 3) $$E\left(f\left(x\right)\right)=\int_{D}f\left(x\right)g\left(x\right)dx=\int_{D}\frac{f\left(x\right)g\left(x\right)}{q\left(x\right)}q\left(x\right)dx=E_{q}\left(\frac{f\left(X\right)g\left(X\right)}{q\left(X\right)}\right)\text{,}$$

where $E_{q}\left(\cdot \right)$ represents expectation for X∼q, w=g(x)/q(x) is the importance weight, q(x) represents the ISD function, and q is referred to as the importance distribution.

By sampling n instances from q, the expectation can be estimated using the sample mean:

(Equation 4) $$\hat{E}\left(f\left(x\right)\right)=\frac{1}{n}\sum_{i=1}^{n}\frac{f\left(X_{i}\right)g\left(X_{i}\right)}{q\left(X_{i}\right)},{\;X}_{i}∼q.$$

Each instance from q is weighted by the importance weight to keep the unbiasedness. It is important to note that, in the process of substituting q for g during the sampling, a vital requirement is that q(x)>0 whenever f(x)g(x)≠0.

Then, the variance of probability estimation (VPE) is given by:

(Equation 5) $${\hat{\sigma }}_{q}^{2}=\frac{1}{n}\sum_{i=1}^{n}{\left(\frac{f\left(X_{i}\right)g\left(X_{i}\right)}{q\left(X_{i}\right)}-\hat{E}\left(f\left(x\right)\right)\right)}^{2},$$

where a high VPE indicates greater deviation of data points from the mean, resulting in increased instability in the estimated probability.

### The proposed GAE-IS method

The proposed GAE-IS method focuses on estimating the probability of extreme failure scenarios involving the failure of multiple link combinations in a transportation network. Each failed link may experience a partial decrease in its capacity, leading to an increase in the ATT within the network. If the ATT of a specific failure scenario exceeds the threshold $\theta_{e}$, this scenario is classified as an extreme failure scenario:

(Equation 6) $${f\left(x\right)=I}_{x}\left\{ATT>\theta_{e}\right\}=\left\{ \begin{array}{c} \;1\;ATT>\theta_{e}\\ \;0\;ATT\leq \theta_{e} \end{array}\right.,$$

where x denotes the variables of the link states in a specific failure scenario, $x=\left(s_{l_{1}},s_{l_{2}},\ldots ,s_{l_{N}}\right)$, $s_{l_{i}}$ represents the state of link $l_{i}$, with a value of 1 indicating failure and a value of 0 indicating normal operation, and N represents the total number of links. In a failure scenario where the link state is x, if the ATT exceeds the threshold $\theta_{e}$, the indicator function, $I_{x}\left\{ATT>\theta_{e}\right\}$, takes the value of 1; otherwise, it is 0.

The objective is to estimate the value of $P\left(ATT>\theta_{e}\right)$, denoted as p, which represents the probability of extreme failure scenarios in the network. The probability p can be calculated as follows:

(Equation 7) $$p=\sum_{x\in X}I_{x}\left\{ATT>\theta_{e}\right\}g\left(x\right)\text{,}$$

where X represents the feasible domain of x, encompassing all possible link state vectors. The term g(x) represents the probability density function of failure scenarios.

For a network with N links, each of which has a mutually independent structural failure probability τ (0<τ≤1), the probability density function g(x) of a failure scenario can be calculated as follows, where z(s) represents the probability density function of the link state:

(Equation 8) $$g\left(x\right)=\prod_{i=1}^{N}z\left(s_{l_{i}}\right),\forall x\in X\text{,}$$

(Equation 9) $$z\left(s_{l_{i}}\right)=s_{l_{i}}\tau_{l_{i}}+\left(1-s_{l_{i}}\right)\left({1-\tau }_{l_{i}}\right)\text{.}$$

Due to the impracticality of exhaustively enumerating all possible failure scenarios, the probability p is estimated through sampling techniques. In the proposed methodology, we utilize the criticality assessor to estimate the likelihood of link $l_{i}$ appearing in the set of failed links in risk scenarios, as a measure of link criticality. The modified criticality is denoted as $c_{l_{i}}$ ($c_{l_{i}}>0$). We take $c_{l_{i}}$ as the adjustment coefficient for the structural failure probability of the link in the ISD function to sample failure scenarios using GAE-IS. Then, the ISD function q(x) for a failure scenario is calculated as follows, where d(s) represents the ISD function of the link state:

(Equation 10) $$q\left(x\right)=\prod_{i=1}^{N}d\left(s_{l_{i}}\right),\forall x\in X\text{,}$$

(Equation 11) $$d\left(s_{l_{i}}\right)=s_{l_{i}}\varphi \left(c_{l_{i}}\tau_{l_{i}}\right)+\left(1-s_{l_{i}}\right)\left({1-c_{l_{i}}\tau }_{l_{i}}\right)\text{,}$$

(Equation 12) $$\varphi \left(c_{l_{i}}\tau_{l_{i}}\right)=\min\left(c_{l_{i}}\tau_{l_{i}},1\right)\text{.}$$

By sampling n instances from the distribution q and substituting Equation 6, Equation 8, and Equation 10 into Equation 4, an estimation of the probability p under q(x) is given by:

(Equation 13) $${\hat{p}}_{q}=\frac{1}{n}\sum_{i=1}^{n}\frac{I_{x_{i}}\left\{ATT>\theta_{e}\right\}g\left(x_{i}\right)}{q\left(x_{i}\right)},x_{i}∼q\text{.}$$

To validate the sampling efficiency of the proposed GAE-IS method, we measure the reduction in VPE achieved by GAE-IS compared to crude Monte Carlo, with the same sample size. In the case of crude Monte Carlo, where samples follow a Bernoulli distribution, the VPE can be calculated as p(1−p). Then, we can obtain the variance reduction ratio as follows:

(Equation 14) $$R=\frac{{\hat{p}}_{q}\left(1-{\hat{p}}_{q}\right)}{{\hat{\sigma }}_{q}^{2}}=\frac{{\hat{p}}_{q}\left(1-{\hat{p}}_{q}\right)/n}{{\text{se}\left({\hat{p}}_{q}\right)}^{2}}\text{,}$$

where ${\hat{\sigma }}_{q}^{2}$ is the VPE in the GAE-IS method and ${\text{se}\left({\hat{p}}_{q}\right)}^{2}$ is the sampling variance of ${\hat{p}}_{q}\text{,}$ with the sampling variance calculated as follows:

(Equation 15) $${\text{se}\left({\hat{p}}_{q}\right)}^{2}=\frac{{\hat{\sigma }}_{q}^{2}}{n}=\frac{1}{n^{2}}\sum_{i=1}^{n}{\left(\frac{I_{x_{i}}\left\{ATT>\theta_{e}\right\}g\left(x_{i}\right)}{q\left(x_{i}\right)}-{\hat{p}}_{q}\right)}^{2}\text{.}$$

In other words, GAE-IS achieves an efficiency improvement by a factor of R compared to crude Monte Carlo within the given sample size.

### Case study networks and data preparation

The performance of the proposed methodology is demonstrated on several real road transportation networks: Berlin-Mitte-Prenzlauerberg-Friedrichshain-Center (BMPFC) in Germany, the northern part of Gold Coast (NGC) in Australia, and Anaheim and Chicago-Sketch (CS) in the United States. The BMPFC, NGC, and Anaheim networks each cover a portion of the city, whereas the CS network covers the whole city. Network configuration parameters are detailed in Table 2.

**Table 2:** Experimental network settings

Network	Berlin		Chicago	
	Berlin-Friedrichshain (for training)	BMPFC	Partial CS (for training)	CS
Number of nodes	224	975	219	933
Original number of links	523	2,184	638	2,950
Number of links after removing extra connector links	387	1,607	638	2,950
Number of zones	23	98	77	387
Number of OD pairs	506	9,505	5,929	142,890
Number of trips	11,205	23,648	362,858	1,260,907
Initial ATT	82.30 (s)	117.81 (s)	9.83 (min)	14.43 (min)
Initial c-link capacity (veh/h)	999,999	999,999	49,500	49,500
Modified c-link capacity (veh/h)	7,000	7,000	25,000	25,000

Network	Anaheim		Northern part of Gold Coast	
	Partial Anaheim (for training)	Anaheim	Partial NGC (for training)	NGC
Number of nodes	138	416	281	1,051
Number of links	294	914	592	2,417
Number of zones	15	38	67	249
Number of OD pairs	210	1,406	4,489	62,001
Number of trips	12,166	104,694	6,375	23,443
Initial ATT	10.87 (min)	12.87 (min)	4.77 (min)	7.13 (min)

In our study, we incorporate the capacity of road segments as the edge attribute. In the original network configurations, there are some spurious road segments, referred to as connector links (c-links), which connect each zone centroid to the surrounding links of that zone. These connector links have disproportionately high capacities and a travel time of zero. To ensure balanced message passing and to prevent the model from overly prioritizing nodes or links with exceptionally high edge attributes, we adjust the capacity for connector links that have significantly higher capacity compared to regular links. For the BMPFC network, the capacity of the connector links is set to 2.5 times the maximum capacity of regular links. In the CS network, which has a broader range of link capacities, the capacity of connector links is set to 5 times the median capacity of the links. The road networks of Anaheim and NGC do not include connector links, and therefore no preprocessing is required. To conclude, the introduction of scaling factors is intended to address the network configuration deficiencies, which do not affect the performance evaluation of the proposed methodology.

### Experimental setup

The parameter settings of applying GAE-IS for sampling failure scenarios in all networks, along with hyperparameter settings for the criticality assessors, are shown in Table 3. Figure S1 illustrates the loss function values and the corresponding VPE for extreme failure scenarios across different epochs. The cross-referencing between the loss function curve and the VPE curve helps us determine the stopping epochs for the training process, where both the loss function value and the VPE, reflecting the sample variance of GAE-IS, converge.

**Table 3:** Experimental parameter settings

Parameters/hyperparameters		BMPFC	Anaheim	NGC	CS
GAE-IS	τ	0.1	0.1	0.05	seismic-based (mean: 0.0156)
	ϵ	0.1			
	$N_{t}$	1 $\times 10^{4}$			
	ρ	10%			
	η	0.15			
	capacity decrease	50%			
Criticality assessors	output channels of conv layers	40, 20			
	Output channels of FC layers	20			
	activation function for each layer	ReLU			
	loss function	SmoothL1Loss			
	optimizer	Adam			
	learning rate	0.002			
	weight decay	0.0001			
	epoch	300	200	100	200

All experiments were conducted on a computer equipped with 64 GB of RAM and an Intel Core i7-11700K processor (3.6 GHz) and running Ubuntu 18.04.1 LTS.

### Details for feature perturbation experiments

**Figure 3:**
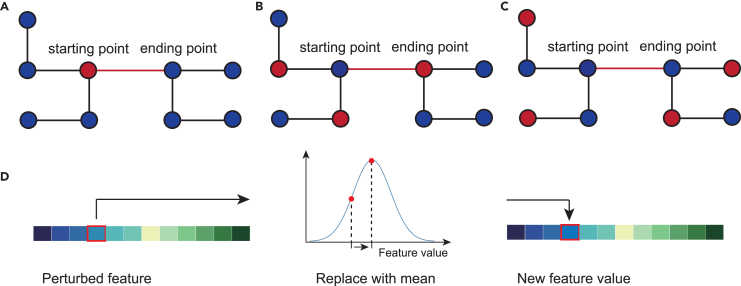
Schematic diagram of the feature perturbation experiment (A–C) The diagrams illustrate the feature perturbation at: (A) the starting point, (B) the one-hop neighbors of the starting point, and (C) the two-hop neighbors of the starting point. (D) The perturbation method involves replacing the specified feature dimension of the perturbed node with the mean value of that feature dimension across all nodes in the network. The perturbation method for the ending point and its one-hop and two-hop neighbors is the same as that for the starting point.

To further investigate the features that significantly contribute to the estimation of criticality of links, we conducted feature perturbation experiments^52^ on node features. Since the criticality assessor comprises two graph neural network layers, the results were calculated by two-hop neighbors of the two endpoints of each link. Therefore, for each link, we perturbed the features of the nodes at the link’s starting point and its one-hop and two-hop neighbors (as shown in Figure 3) as well as the link’s ending point and its one-hop and two-hop neighbors separately. The concatenation of these nodes’ feature vectors is termed as the extended node feature vector for perturbation experiments. Specifically, we replaced these features with the mean value of that feature across all nodes. The impact of perturbing each dimension of extended node feature vector on the model’s output is quantified by the magnitude of change in the estimated link likelihood, denoted as Δh(EV), and calculated as follows:

(Equation 16) $$\Delta h_{l_{i}}\left({EV}_{jk}^{l_{i}}\right)=\frac{\left|{\hat{h}}_{l_{i}}^{p}\left({EV}_{jk}^{l_{i}}\right)-{\hat{h}}_{l_{i}}\right|}{{\hat{h}}_{l_{i}}}\text{,}$$

where ${EV}_{jk}^{l_{i}}$ represents the k-th feature of the j-th node in the extended node feature vector of link $l_{i}$, ${\hat{h}}_{l_{i}}$ represents the original estimated likelihood for link $l_{i}$, and ${\hat{h}}_{l_{i}}^{p}\left({EV}_{jk}^{l_{i}}\right)$ represents the estimated likelihood for link $l_{i}$ after perturbing ${EV}_{jk}^{l_{i}}$. When a starting point or ending point has multiple one-hop or two-hop neighbors, the features of each neighbor are perturbed individually, and the mean value of Δh(EV) for that range of neighbors is calculated to assess the impact. Δh(EV) quantifies the relative impact of feature variations on the model’s estimation of link criticality. A larger value of Δh(EV) indicates a greater importance of the feature.

It is important to note that, according to the conservation law^53^ of traffic flow, when a node serves as an intersection, its inflow is equal to its outflow. As a result, any perturbation in the inflow at an intersection node will correspondingly affect its outflow and vice versa. If a node is an OD point, the perturbations in inflow and outflow are independent of each other. Since the remaining capacity of a node is the difference between its capacity and its flow (as presented in Table 1), when the node’s inflow/outflow is perturbed, the remaining capacity of the node as an ending point/starting point will also change accordingly. Similarly, perturbing the remaining capacity will lead to changes in the inflow/outflow.

## Results

### Improved efficiency in sampling extreme failure scenarios using GAE-IS

We evaluated the performance of the proposed GAE-IS method in road transportation systems. The road networks in BMPFC, Anaheim, and NGC are taken as case study networks with homogeneous link failure probabilities. The performance of the GAE-IS under heterogeneous link failure probabilities will be analyzed in a subsequent subsection.

**Figure 4:**
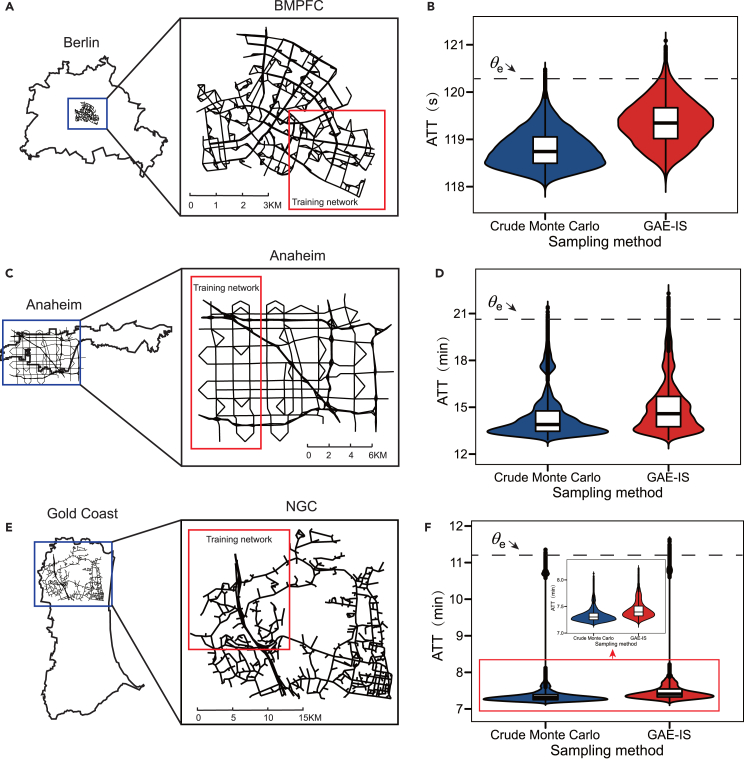
Sampling results for road networks in BMPFC, Anaheim, and NGC (A) Schematic diagram of Berlin’s city boundaries and the BMPFC network. (B) Violin plot of the ATT distribution for $2\times 10^{4}$ failure scenarios sampled from the BMPFC network. (C) Schematic diagram of Anaheim’s city boundaries and the Anaheim network. (D) ATT distribution for $2\times 10^{4}$ failure scenarios sampled from the Anaheim network. (E) Schematic diagram of Gold Coast’s city boundaries and the NGC network. (F) ATT distribution for $2\times 10^{4}$ failure scenarios sampled from the NGC network.

When compared to crude Monte Carlo simulations conducted with the same sample size, GAE-IS demonstrates superior capability in identifying extreme failure scenarios that lead to greater system performance degradation (as shown in Figures 4B, 4D, and 4F). To quantify the reduction in VPE and the sampling efficiency improvement achieved by GAE-IS, the variance reduction ratio R was computed. The case study results show that GAE-IS provides a significant reduction in VPE, indicating enhanced sampling quality compared to crude Monte Carlo. This improvement is particularly pronounced in extreme failure scenarios with higher thresholds $\theta_{e}$, where sampling efficiency increases by 82, 45, and 139 times for the BMPFC, Anaheim, and NGC networks, respectively (as shown in Table 4). Additionally, the criticality assessor, trained on sub-networks, effectively supports the GAE-IS process when applied to the larger-scale original road networks. On the three case networks, performing presampling on the sub-networks saves 12, 9, and 19 times the computational time, respectively, compared to performing presampling on the original network under the same computational resource setting. These findings demonstrate the transferability of the proposed criticality assessor and highlight the feasibility and effectiveness of using presampling on sub-networks to obtain ISD for large-scale networks at much lower computational cost, taking advantage of the GAE-IS method.

**Table 4:** Sampling results of GAE-IS in case studies

Network	Presampling time (hour)		Sample size	$\theta_{e}$	$\hat{p}$	VPE	R
	Sub-network	Large-scale network					
BMPFC	3.92	46.00	2 $\times 10^{4}$	120.30	7.40 $\times 10^{-4}$	9.91 $\times 10^{-4}$	1
				120.75	1.44 $\times 10^{-6}$	1.76 $\times 10^{-8}$	82
Anaheim	1.98	18.67	2 $\times 10^{4}$	20.47	5.84 $\times 10^{-4}$	9.13 $\times 10^{-4}$	1
				21.89	2.88 $\times 10^{-6}$	6.40 $\times 10^{-8}$	45
NGC	6.64	125.00	2 $\times 10^{4}$	11.28	5.12 $\times 10^{-5}$	9.48 $\times 10^{-6}$	5
				11.58	4.60 $\times 10^{-7}$	3.31 $\times 10^{-9}$	139

### Sensitivity analysis of the GAE-IS performance

The parameter η determines how many links in the network are considered critical in importance sampling. Sensitivity analyses are conducted on case networks to assess the impact of different settings of η on the performance of GAE-IS.

**Figure 5:**
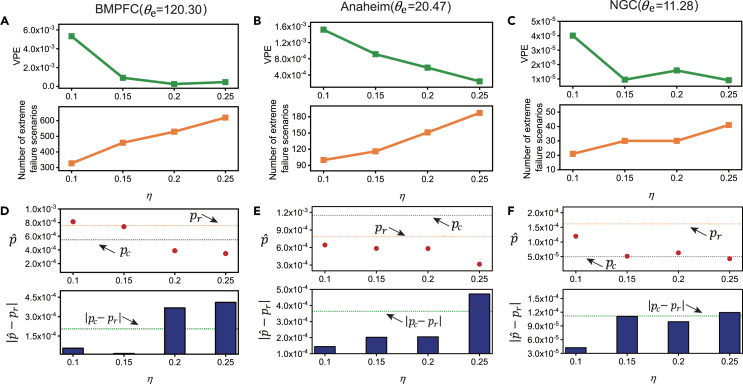
Sampling results for case networks with four different η settings, i.e., 0.1, 0.15, 0.2, and 0.25 For each parameter setting, $2\times 10^{4}$ failure scenarios were sampled. (A) Number of extreme failure scenarios sampled and the VPE for the BMPFC network. (B) Number of extreme failure scenarios sampled and the VPE for the Anaheim network. (C) Number of extreme failure scenarios sampled and the VPE for the NGC network. (D) Estimated probabilities of extreme failure scenarios and errors compared to the reference probability for the BMPFC network. (E) Estimated probabilities of extreme failure scenarios and errors compared to the reference probability for the Anaheim network. (F) Estimated probabilities of extreme failure scenarios and errors compared to the reference probability for the NGC network. Red dots represent probabilities estimated via GAE-IS, orange dashed lines denote the reference probability ($p_{r}$) derived through $5\times 10^{5}$ crude Monte Carlo samples, and gray dashed lines indicate the probabilities (denoted as $p_{c}$) estimated from $2\times 10^{4}$ crude Monte Carlo samples. Blue bars show the absolute error between the GAE-IS estimates and the reference probability, while green dashed lines represent the absolute error between the probabilities estimated from $2\times 10^{4}$ crude Monte Carlo samples and the reference probability.

The efficiency and precision of GAE-IS were evaluated according to two metrics, i.e., the number of sampled extreme failure scenarios and the VPE, respectively. Furthermore, to evaluate the accuracy of probability estimation under limited sample sizes, we compared the probabilities estimated by GAE-IS with the reference probability (denoted as $p_{r}$) derived from $5\times 10^{5}$ crude Monte Carlo samples. The results, illustrated in Figures 5A–5C, show that increasing η enhances the ability of GAE-IS to capture more extreme failure scenarios. This increase in η also leads to a reduction in VPE for these scenarios, whereas, when η is set to a lower value, the probability estimates provided by GAE-IS align more closely with the reference probability compared to those with larger η. Moreover, with a lower η setting, GAE-IS also outperforms crude Monte Carlo sampling with an equivalent sample size in terms of probability estimation accuracy, as shown in Figure 5. However, as η increases, the accuracy of the probability estimates deteriorates, with larger discrepancies observed between GAE-IS and the reference probability $p_{r}$, as depicted in Figures 5D–5F. This suggests that, at lower values of η, GAE-IS provides more accurate estimates.

An intriguing observation arises as the parameter η increases: despite a reduction in sample variance, there is an increase in the error of probability estimation. This phenomenon indicates that η has a significant impact on the accuracy of probability estimates when the sample size is limited. This trade-off highlights the complexity of balancing between sample variance and estimation accuracy in the GAE-IS method. As the number of links with adjusted failure probabilities increases, the variance in the importance weights for failure scenarios also grows, leading to larger errors in the estimated probabilities. Moreover, the criticality of each link differs, and its contribution to the variance in the importance weights is also variable. The non-linear cumulative effect of criticality leads to a disproportionate growth in the variance of the importance weights for failure scenarios with the increase in η. Therefore, adjusting the failure probabilities of critical links determined by η rather than all links is essential for achieving more accurate probability estimates. In practice, to achieve more accurate probability estimates for extreme failure scenarios, it is advisable to use a relatively lower η. In our case study networks, η taking the value of 0.1 or 0.15 is effective for achieving this accuracy. If it is required to further mitigate the impact of variance in importance weights on the accuracy of probability estimates, link criticality can be modified by employing scaling factors and constraining value ranges to reduce the fluctuations in the criticality of links. On the other hand, if the goal is to capture more extreme failure scenarios or achieve a smaller VPE, it may be beneficial to increase η, albeit with the understanding that this might introduce larger errors in probability estimation.

### Application of GAE-IS in the context of heterogeneous link failure probabilities

**Figure 6:**
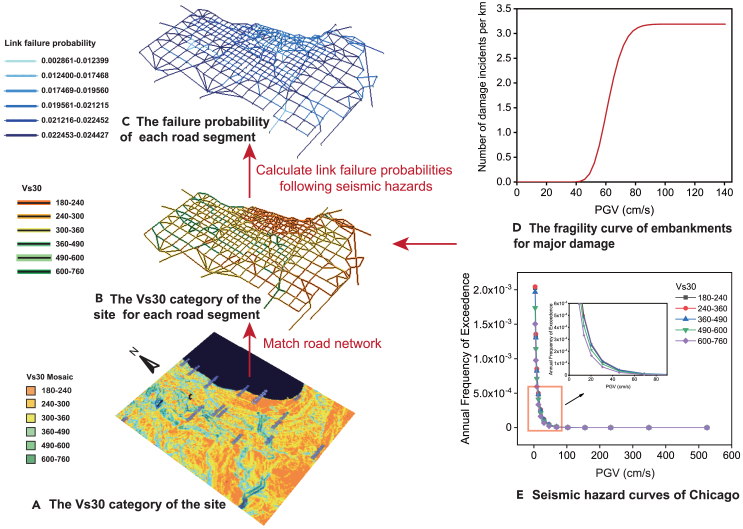
The procedure for estimating link failure probabilities for major damage caused by earthquakes in Chicago

To examine the effectiveness of the proposed GAE-IS methodology, it was applied to the Chicago road network, specifically focusing on the failure probabilities of road segments due to seismic events. Since minor damage generally does not significantly affect road capacity,^54^ this case study focuses on major damage to road segments. The failure probabilities of major damage to road segments were calculated based on the geographical seismic risk and the fragility curve of embankments. First, according to the mosaic-based Vs30 raster data from the Vs30 Map Viewer,^55^ the Vs30 category of the site for each road segment was determined (Figure 6B). By integrating this category with the seismic hazard curve^56^ (Figure 6E), the annual frequency curve of earthquakes exceeding a specified intensity for each road segment was derived. Subsequently, the seismic intensities were discretized, and based on the fragility curve^57^ (Figure 6D), the number of major damage events per unit length of road segment within each intensity range was obtained. Leveraging these data, and assuming that earthquake occurrences follow a Poisson distribution, we calculated the conditional probability of failure for each road segment (Figure 6C). Detailed calculation methods can be found in Appendix C in the supplemental methods. Although we presented the risk of damage to only the CS network under seismic hazards, the estimation method for link failure probabilities can also be applied to other case networks. Ultimately, the failure probabilities of the road segments in the CS network were heterogeneous. However, in the CS network, the workflow of GAE-IS remained the same as in a network with homogeneous link failure probabilities, except that the structural failure probability, τ, was replaced by heterogeneous link failure probabilities.

**Figure 7:**
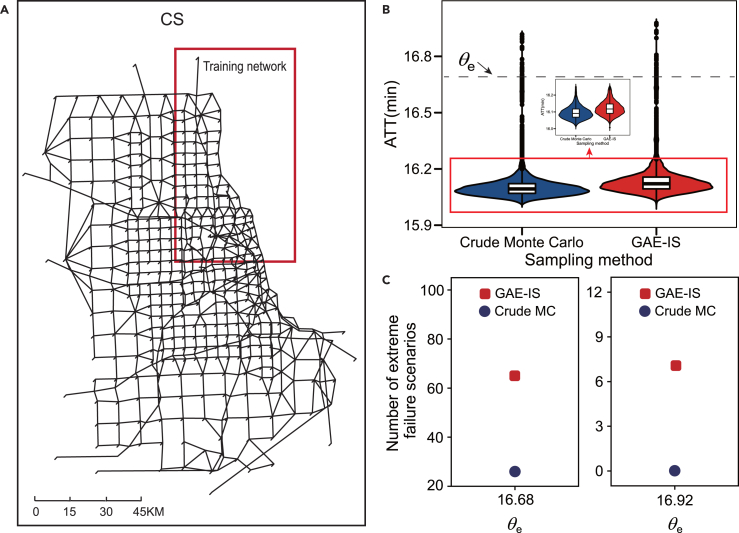
Sampling results on the Chicago-Sketch road network (A) Schematic diagram of the CS network. (B) The violin plot shows the distribution of ATT for $1\times 10^{4}$ failure scenarios sampled for the CS network. (C) The number of extreme failure scenarios sampled by GAE-IS and crude Monte Carlo.

The results demonstrate that GAE-IS significantly outperforms crude Monte Carlo in sampling extreme failure scenarios with the same sample size (Figure 7). GAE-IS improved the sampling efficiency for extreme failure scenarios on the CS network by 2–40 times compared to crude Monte Carlo (as shown in Table 5). In addition, performing presampling on the sub-network took only 1/32 of the computational time required for presampling on the original network. These findings confirm that GAE-IS is both feasible and effective even in scenarios involving spatially heterogeneous link failure probabilities.

**Table 5:** Sampling results of GAE-IS in Chicago-Sketch road network

Network	Presampling time (hour)		Sample size	$\theta_{e}$	$\hat{p}$	VPE	R
	Sub-network	Large-scale network					
CS	15.48	493.37	1 $\times 10^{4}$	16.68	8.64 $\times 10^{-4}$	4.60 $\times 10^{-4}$	2
			1 $\times 10^{4}$	16.92	9.55 $\times 10^{-6}$	2.37 $\times 10^{-7}$	40

The GAE-IS methodology effectively decouples component criticality from vulnerability, allowing for straightforward updates to the ISD function in response to changes in seismic risk. Given a different seismic hazardous event, this process merely requires the integration of new link failure probabilities alongside their associated criticality.

While our primary focus has been on earthquakes, the framework of GAE-IS is versatile and can be applied to other disaster scenarios as well. Similar to updates necessitated by changes in seismic risk, adapting to different types of disasters does not require redoing presampling either. Instead, it suffices to replace the vulnerability distribution accordingly.

As a result, GAE-IS significantly reduces the cost of importance sampling for different disruptive events on the same network, making it a valuable tool for risk assessment and management.

### Features affecting link criticality in networks

**Figure 8:**
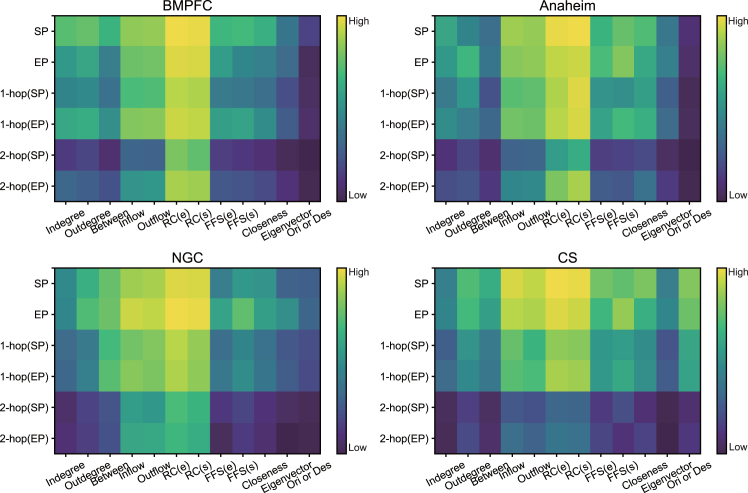
Feature perturbation results for criticality assessors across different road networks, illustrating the contribution of different features to the criticality of road segments Warmer colors indicate higher importance, while cooler colors represent lower importance. “SP” refers to the starting point, “EP” denotes the ending point, “1-hop (SP)” represents the one-hop neighbors of the starting point, and “2-hop (SP)” refers to the two-hop neighbors of the starting point. Similarly, “1-hop (EP)” and “2-hop (EP)” represent the one-hop and two-hop neighbors of the ending point, respectively. “Between” refers to betweenness centrality, “RC (e/s)” refers to the remaining capacity of the node as an ending/starting point, and “FFS (e/s)” refers to the free-flow speed of the node as an ending/starting point.

The average results of feature perturbation for all links are shown in Figure 8. The results demonstrate that the remaining capacity of nodes is the most critical feature across all road networks, followed by the flow passing through the node, which ranks as the second most important feature. This pattern is consistent across different road networks, indicating that both the node capacity and the assigned traffic flow passing through the node are essential factors in identifying critical links for network functionality. Although the quantitative impact of these key factors on the estimated link likelihoods varies across different networks, this observation reinforces confidence in the potential to enhance the transferability of the GAE-IS method across various networks.

## Discussion

This study proposes GAE-IS to estimate the probabilities of extreme failure scenarios in large-scale infrastructure networks, focusing on transportation systems to build up this methodology and investigate its performance. Central to our methodology is the development of the criticality assessor—a graph learning model that integrates topological centrality metrics with traffic flow attributes of network components to derive ISD functions. One of the key advantages of GAE-IS is its significantly lower computational cost for sampling extreme failure scenarios that lead to substantial performance degradation in network-structured infrastructure systems. This computational efficiency is largely attributed to the transferability of the criticality assessor. Additionally, the workflow of GAE-IS facilitates the decoupling of link criticality from failure risk. This separation allows for the integration of vulnerability distributions with link criticality distribution obtained through the criticality assessor. As a result, it facilitates a more streamlined approach to efficient acquisition of ISD functions, eliminating the need for extensive presampling and retraining of the model across various vulnerability distributions.

Extensive experiments demonstrate the effectiveness and efficiency of the proposed GAE-IS method for sampling extreme failure scenarios in road transportation networks. Experimental results on road networks with homogeneous link failure probabilities, such as those in Berlin, Anaheim, and the NGC, as well as on the Chicago road network with heterogeneous link failure probabilities under seismic risk, demonstrate that the GAE-IS method effectively captures more extreme failure scenarios. With a given sample size, it reduces the VPE and enhances sampling efficiency by one to two orders of magnitude, while also providing more accurate extreme failure probability estimates compared to crude Monte Carlo. Hence, the proposed GAE-IS method demonstrates significant promise for assessing the resilience of infrastructure networks from the perspective of extreme value statistics. Its design allows for straightforward extension to various types of infrastructure systems by adjusting the presampling of failure scenarios and the feature extraction for components in the objective network. For instance, future efforts can apply the proposed GAE-IS method to evaluate the extreme failure probability under a specific type of hazard for power grids, water distribution systems, etc. This flexibility enhances the method’s applicability across different types of networks and research contexts.

The current GAE-IS is primarily applicable to infrastructure systems that have reached a mature stage, where the network’s functionality and structure are well developed and the demand remains stable. If the system is still in the planning, construction, or operational testing phase, leading to significant changes in the network’s topology, or if shifts in factors such as population structure, economic development, or lifestyle alter the spatial travel distribution pattern dramatically, retraining the model becomes necessary. This is because the criticality of network links may change as a result. However, the feature perturbation experiments for the criticality assessors reveal a consistent pattern in how node features, including topological centrality metrics and traffic flow attributes, contribute to link criticality. This phenomenon motivates further exploration of the transferability of GAE-IS across different networks with distinct topological structures and demand distribution patterns, such as networks that are undergoing development, as well as networks in different cities. Addressing this challenge and developing methods to build further transferability represent important directions for future research.

In conclusion, GAE-IS proves to be an effective and efficient tool for evaluating infrastructure system resilience. It is particularly applicable for systems that require substantial computational effort for their performance evaluation, especially in the context of analyzing extreme scenarios. The advantages of GAE-IS suggest promising applications in several key areas, including the design and operation of resilient infrastructure systems, the development of resilient cities, and the advancement of sustainable communities. Future research could explore further optimizations of the GAE-IS method to enhance its performance in diverse scenarios and expand its application across various infrastructure systems, such as water distribution networks, power grids, and communication systems. One promising direction involves refining the selection criteria of the sub-network for training the criticality assessor. Currently, approximately one-fourth to one-third of the original networks are randomly selected to serve as training networks for criticality assessors. We aim to explore a more systematic approach for sub-network selection, such as investigating the structural and functional similarities between sub-networks and the overall network, which could provide a theoretical basis for selecting training networks. By choosing more representative sub-networks that better capture the characteristics of the entire network, the performance of the GAE-IS method can be further enhanced.

## Resource availability

### Lead contact

Requests for further information and resources should be directed to and will be fulfilled by the lead contact, Tingting Zhao (ttzhao@bjtu.edu.cn).

### Materials availability

This study did not generate new materials.

### Data and code availability

The data for transportation networks can be accessed from the open-source transportation network dataset: https://github.com/bstabler/TransportationNetworks. The mosaic-based Vs30 raster data were obtained from the Vs30 Map Viewer,^55^ while the seismic hazard curves were sourced from the USGS Earthquake Hazard Toolbox.^56^ The code for GAE-IS is available at figshare.^58^

## Acknowledgments

The authors disclose support for the research of this work from the 10.13039/501100001809National Natural Science Foundation of China (72288101, 72201028, 72242102, and 72091513) and the USA National Science Foundation (2047488).

## Author contributions

Conceptualization, T.Z., Z.G., and J.G.; data curation, M.G., T.Z., J.G., and X.M.; formal analysis, T.Z. and M.G.; funding acquisition, Z.G. and T.Z.; investigation, T.Z. and M.G.; methodology, T.Z., Z.G., and M.G.; project administration, resources, and supervision, Z.G. and T.Z.; visualization, T.Z. and M.G.; writing – original draft, T.Z. and M.G.; writing – review & editing, Z.G. and J.G.

## Declaration of interests

The authors declare no competing interests.

## Supplemental information

Document S1. Figure S1 and supplemental methods

Document S2. Article plus supplemental information
