# Supplementary material for: Quantifying extreme failure scenarios in transportation systems with graph learning
Source: Patterns (N Y). 2025 Mar 14;6(4):101209. doi: 10.1016/j.patter.2025.101209 (PMC12010444; doi:10.1016/j.patter.2025.101209)
Supplement: Document S1. Figure S1 and supplemental methods [file mmc1.pdf]

Patterns, Volume *6*

Supplemental information

Quantifying extreme failure scenarios in transportation systems with graph learning

Mingxue Guo, Tingting Zhao, Jianxi Gao, Xin Meng, and Ziyou Gao

Supplemental methods

Appendix A: The formulation of User Equilibrium

This study takes the Average Travel Time (ATT) of the transportation network as the performance indicator. To calculate the ATT, we utilize User Equilibrium[1] to assign traffic demand in the network (Equations S1–S4). User Equilibrium (UE) assumes that road users have complete knowledge of the traffic conditions in the network and strive to choose paths with minimum travel cost. When the network reaches the state of user equilibrium, the travel times of all used paths for each origin-destination pair are equalized and minimized, and no user can unilaterally reduce their travel time by changing their route. The computation method of UE exemplifies the self-adaptability of travelers to congestion in the road network.

$$\min Z_{UE}(x) = \sum_i \int_0^{x_{l_i}} t_{l_i}(x)dx \quad \text{(Equation S1)}$$

$$\text{s.t. } \sum_{k\in K^\omega} f_k^\omega = u^\omega, \ \ \forall\omega \in W \quad \text{(Equation S2)}$$

$$x_{l_i} = \sum_\omega \sum_{k\epsilon K^\omega} \varpi_{l_i k}^\omega f_k^\omega \quad \text{(Equation S3)}$$

$$f_k^\omega \geq 0, \forall k \in K^\omega, \omega \in W \quad \text{(Equation S4)}$$

where x_{l_i} represents the volume on road segment l_i; $t_{l_i}(x)$ is the cost function for road segment l_i, which in this case is the BPR function; f_k^ω denotes the flow on path k for the origin-destination (OD) pair ω; u^ω represents the total traffic demand for the OD pair ω; W denotes the set of all OD pairs, and K^ω represents the set of feasible paths for OD pair ω; $\varpi_{l_i k}^\omega$ is a binary variable that takes the value 1 if path k for OD pair ω passes through road segment l_i, and 0 otherwise. Equation S2 guarantees that the sum of path flows between an origin-destination (OD) pair equals the total traffic demand for that OD pair. Equation S3 denotes the composition relationship between a link flow and path flows going through this link. Equation S4 ensures that all path flows remain non-negative.

Appendix B: Supplemental description of the GAE-IS workflow

If each Link l_i in the target network has an expected structural failure probability denoted as $\tau_{l_i}(0 < \tau_{l_i} \leq 1)$, we employ the following approach for importance sampling.

(1) Assuming that each link in the training network has a homogeneous hypothetical failure probability ϵ $(0 < \epsilon < 1)$, the crude Monte Carlo is employed to randomly sample N_t network failure scenarios with a link failure probability of ϵ as the original sample set.

(2) A predetermined percentage (the ρ percentage) of original samples that demonstrate substantial degradation in network performance are selected as risk scenarios. The corresponding risk scenario threshold for Average Travel Time (ATT) is denoted as θ_r.

(3) The weighted frequency of link occurrence within the failed link set in risk scenarios and the likelihood of links are computed as follows.

$$F_{l_i} = \sum_{k=1}^{\rho N_t} \frac{\epsilon N_a}{N_{fk}} \delta_{l_i k} \quad \text{(Equation S5)}$$

$$h_{l_i} = \frac{F_{l_i}}{E(F)} = \frac{F_{l_i}}{\rho N_t \epsilon} \qquad \text{(Equation S6)}$$

where F_{l_i} represents the weighted frequency of Link l_i occurring within the failed link set in risk scenarios; N_{fk} represents the number of failed links in risk scenario k; ϵ is the hypothetical failure probability of links in the training network, N_a represents the number of links in the training network, and ϵN_a represents the expected number of failed links in a given scenario; $\delta_{l_i k}$ is a binary variable, if Link l_i belongs to the failed link set in risk scenario k, $\delta_{l_i k}$ takes the value of 1; otherwise, it is 0; h_{l_i} ($h_{l_i} > 0$) represents the likelihood of Link l_i appearing in the failed link set in risk scenarios; $E(F)$ represents the expected number of occurrences of a link within the failed link set in risk scenarios.

(4) Using h_{l_i} to adjust the failure probability of links, where each link now has a failure probability of $\min(h_{l_i}\epsilon, 1)$. Sampling N_t network failure scenarios based on $\min(h_{l_i}\epsilon, 1)$ to create a new sample set.

(5) Select top ρ of the new sample set that demonstrate substantial degradation in network performance as risk scenarios. If the corresponding risk scenario threshold θ_r' of the new sample set exceeds the extreme failure scenario threshold θ_e, execute Step (6); otherwise, return to Step (3).

(6) Train Criticality Assessor with h_{l_i} as the link label. A trained Criticality Assessor can be transferred to the target network and outputs the estimated $\hat{h}_{l_i}$ for the target network.

(7) The $\hat{h}_{l_i}$ of all links is sorted in descending order. The top η proportion of links are critical links, and the bottom η proportion of links are non-critical links. Then, $\hat{h}_{l_i}$ will be modified as follows:

$$c_{l_i} = \begin{cases} \frac{2\eta N \hat{h}_{l_i}}{\sum_{l_i \in B} \hat{h}_{l_i}} & l_i \in B \\ 1 & l_i \notin B \end{cases} \qquad \text{(Equation S7)}$$

where c_{l_i} is the modified $\hat{h}_{l_i}$, N is the number of links in the target network, B is the set of critical and non-critical links.

(8) Based on c_{l_i} and τ_{l_i}, perform importance sampling to generate failure scenarios and estimate the probability of extreme failure scenarios in the target network.

Appendix C: The seismic failure probability of road segments in Chicago road network

The average shear wave velocity for the top 30 meters (Vs30) is an important parameter in seismic engineering. It can be used to evaluate the amplification and attenuation characteristics of seismic waves propagating underground[2,3], which is crucial for seismic risk analysis. According to Vs30, the site can be divided into different categories, and determining the site category is a key step in analyzing site seismic effects. Therefore, Vs30 is widely applied as an important site parameter in various fields of seismic engineering, such as seismic risk assessment and seismic design of buildings[4]. In this study, the Vs30 data for Chicago is derived from the mosaic-based Vs30 raster data provided by the Vs30 Map Viewer[5] of the United States Geological Survey (USGS). The Vs30 category of the site for each road segment is determined

by the predominant Vs30 category among the raster cells traversed by the segment (Figure 6B in the main text).

USGS Earthquake Hazard Toolbox is the "web applications for querying and computing hazard from USGS national seismic hazard models" (https://earthquake.usgs.gov/nshmp/). It provides seismic hazard curves for different site categories within the United States. The seismic hazard curve for Chicago is retrieved as shown in Figure 6E in the main text. The horizontal axis represents the peak ground velocity (PGV in cm/s), indicating the maximum velocity of ground motion during an earthquake. It is a parameter that represents the intensity of earthquakes and can be used to assess the impact of earthquakes on buildings and structures. The vertical axis represents the annual frequency of earthquakes exceeding a specific PGV. Based on the Vs30 category of the site for each road segment, the corresponding seismic hazard curve can be obtained.

The seismic damage to urban roads is usually caused by ground displacement. Due to the lack of data on possible ground deformation in Chicago, we use the vulnerability curve of road embankments to measure seismic damage to roads. Maruyama et al.[6] fitted a vulnerability curve for embankments based on recorded data from actual earthquake disasters. This curve reflects the number of major damage incidents per unit length of the embankment at different PGVs (Figure 6D in the main text). Their study is also documented in the European funded research project "Systemic Seismic Vulnerability and Risk Analysis for Buildings, Lifeline Networks and Infrastructures Safety Gain" (SYNER-G)[7]. In seismology, earthquake occurrences are often regarded as a Poisson process, thus the failure probability ζ of a link can be expressed as the Equation S8[8]:

$$\zeta = 1 - e^{-\lambda \iota} \quad \text{(Equation S8)}$$

where λ represents the number of major damage incidents per unit length of the embankment, which is related to PGV; ι is the length of the link.

We compute failure probabilities for road segments in Chicago under the condition of earthquakes with PGV in the range of 4-525 cm/s. Based on the data points along the seismic hazard curve, PGV has been segmented into 12 intervals. The annual frequency of earthquakes with PGV in a specific interval can be calculated by taking the difference in annual frequency of exceedence between interval endpoints. The number of major damage incidents per unit length of the embankment in each PGV interval is represented by the λ of the median PGV of each interval. Finally, the structural failure probability τ for a road segment under the condition of earthquakes with PGV in the range of 4-525 cm/s can be calculated using the Equation S9.

$$\tau = \frac{\sum_{i=1}^{12} \chi_i \zeta_i}{\sum_{i=1}^{12} \chi_i} \quad \text{(Equation S9)}$$

where χ_i represents the annual frequency of earthquakes with PGV falling within the i-th interval; ζ_i represents the failure probability of a link with PGV falling within the i-th interval.

Moreover, the failure probability of all connector links from the zone centroid to the surrounding links is set to be 0.

Appendix D: Training iteration, loss function and VPE of Criticality Assessor

Figure S1 illustrates the variation trend of the loss function and the VPE for extreme failure scenarios versus number of epochs.

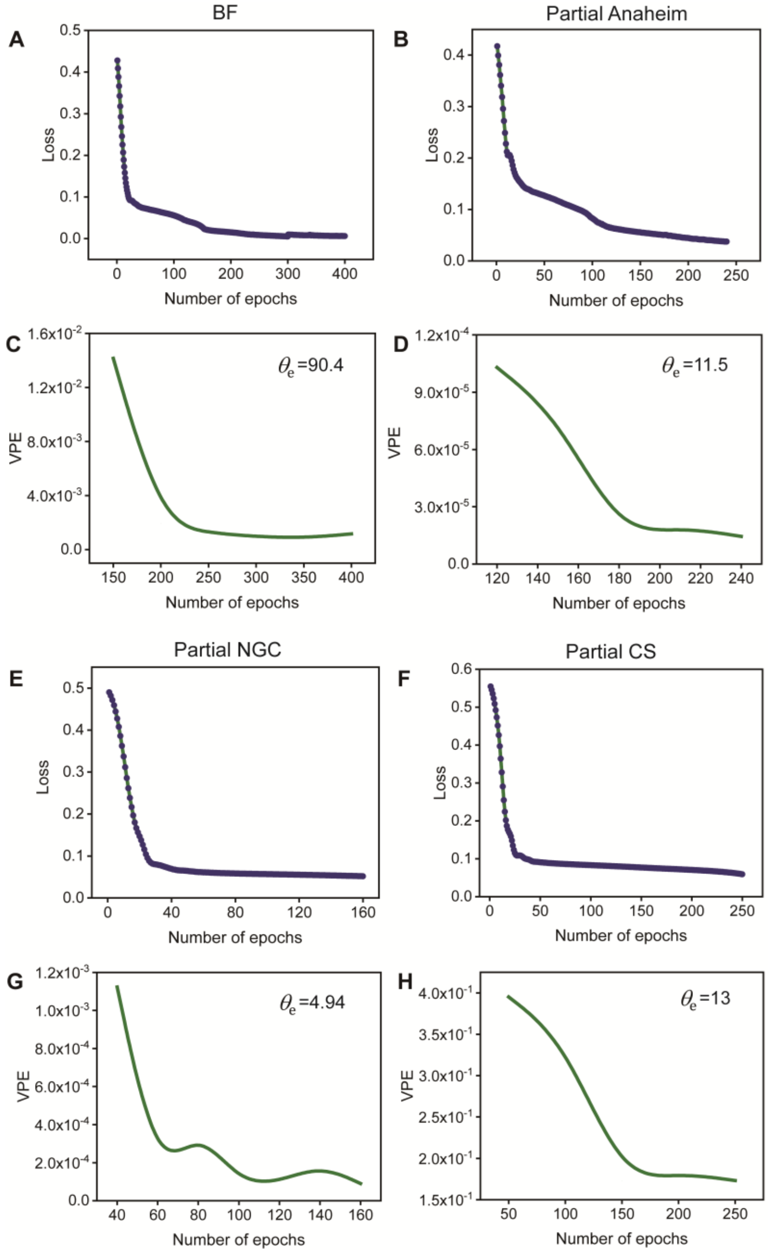

Figure S1: Values of loss function over epochs in the training of Criticality Assessor and the VPE for extreme failure scenarios with respect to each epoch.

(A) Loss function curve when training Criticality Assessor on the BF network.

(B) Loss function curve when training Criticality Assessor on the partial Anaheim network.

(C) VPE of BF network over epochs with the Criticality Assessor trained in (A).

(D) VPE of partial Anaheim network over epochs with the Criticality Assessor trained in (B).

(E) Loss function curve when training Criticality Assessor on partial NGC network.

(F) Loss function curve when training Criticality Assessor on partial CS network.

(G) VPE of partial NGC network over epochs with the Criticality Assessor trained in (E).

(H) VPE of partial CS network over epochs with the Criticality Assessor trained in (F).

Supplemental references

1. Wardrop, J. G (1952). Some theoretical aspects of road traffic research. In ICE Proceedings of Engineering Divisions. pp. 325-378.
2. Alvarado, P., Christiansen, R., Gregori, S. D., and Saez, M. (2020). Evidence of site amplification from ground motion of the last two large crustal earthquakes in central-western Argentina. Nat. Hazards *102*, 1011-1031.
3. Karimzadeh, S., Feizizadeh, B., and Matsuoka, M. (2017). From a GIS-based hybrid site condition map to an earthquake damage assessment in Iran: Methods and trends. Int. J. Disaster Risk Reduc. *22*, 23-36.
4. Abbasnejadfard, M., Bastami, M., Jafari, M. K., and Azadi, A. (2023). Spatial correlation models of VS30 values: A case study of the Tehran region. Eng. Geol. *325*, 107300.
5. Vs30 Map Viewer. https://usgs.maps.arcgis.com/apps/webappviewer/index.html?id=8ac19bc334f747e486550f32837578e1.
6. Maruyama, Y., Yamazaki, F., Mizuno, K., Tsuchiya, Y., and Yogai, H. (2010). Fragility curves for expressway embankments based on damage datasets after recent earthquakes in Japan. Soil Dyn. Earthq. Eng. *30*, 1158-1167.
7. Kaynia, Amir M., Iervolino, I., Taucer F., and Hancilar, U. (2013). Guidelines for deriving seismic fragility functions of elements at risk – Buildings, lifelines, transportation networks and critical facilities. Publications Office of the European Union. https://publications.jrc.ec.europa.eu/repository/bitstream/JRC80561/lbna25880enn.pdf.
8. Wisetjindawat, W., Kermanshah, A., Derrible, S., and Fujita, M. (2017). Stochastic Modeling of Road System Performance during Multihazard Events: Flash Floods and Earthquakes. J. Infrastruct. Syst. *23*, 04017031.
