# Supplementary material for: Quantifying extreme failure scenarios in transportation systems with graph learning
Source: Patterns (N Y). 2025 Mar 14;6(4):101209. doi: 10.1016/j.patter.2025.101209 (PMC12010444; doi:10.1016/j.patter.2025.101209)
Supplement: Document S2. Article plus supplemental information [file mmc2.pdf]

Article

Patterns

# Quantifying extreme failure scenarios in transportation systems with graph learning

## Highlights

- Proposes a sampling framework that decouples component criticality from failure risk
- Introduces a transferable component criticality assessor
- Enhances sampling efficiency for extreme events in large-scale transport systems
- Identifies consistent feature patterns influencing link criticality across networks

## Authors

Mingxue Guo, Tingting Zhao, Jianxi Gao, Xin Meng, Ziyou Gao

## Correspondence

ttzhao@bjtu.edu.cn (T.Z.),
zygao@bjtu.edu.cn (Z.G.)

## In brief

Analyzing extreme events in large-scale engineering systems is computationally expensive due to the rarity of such events. To overcome this, the authors propose the GAE-IS method, which integrates a modified graph autoencoder with cross-entropy-based importance sampling. Their approach significantly enhances sampling efficiency and accuracy in extreme scenario probability estimation, with experiments on road networks from four cities demonstrating improvements by one to two orders of magnitude.

Guo et al., 2025, Patterns *6*, 101209
April 11, 2025 © 2025 The Authors. Published by Elsevier Inc.
https://doi.org/10.1016/j.patter.2025.101209

CellPress

Patterns

CellPress
OPEN ACCESS

Article

# Quantifying extreme failure scenarios in transportation systems with graph learning

Mingxue Guo,[1,3] Tingting Zhao,[1,3,4,*] Jianxi Gao,[2] Xin Meng,[1] and Ziyou Gao[1,*]
[1]School of Systems Science, Beijing Jiaotong University, Beijing 100044, China
[2]Department of Computer Science, Rensselaer Polytechnic Institute, Troy, NY 12180, USA
[3]These authors contributed equally
[4]Lead contact
*Correspondence: ttzhao@bjtu.edu.cn (T.Z.), zygao@bjtu.edu.cn (Z.G.)
https://doi.org/10.1016/j.patter.2025.101209

**THE BIGGER PICTURE** Extreme events, like extreme weather, earthquakes, and other disasters, although rare, can have catastrophic effects on critical infrastructure such as transportation networks and power grids. Predicting the impact of these "black swan" events and estimating the probability of extreme failures has long been a challenge due to the high computational cost of traditional methods. Here, the researchers introduce an approach that leverages graph learning techniques to reduce computational burden and improve the accuracy of extreme failure probability estimation. This method could be applied to evaluate the resilience of diverse urban infrastructures, including power, transportation, and water supply systems.

## SUMMARY

Statistical analysis of extreme events in complex engineering systems is essential for system design and reliability and resilience assessment. Due to the rarity of extreme events and the computational burden of system performance evaluation, estimating the probability of extreme failures is prohibitively expensive. Traditional methods, such as importance sampling, struggle with the high cost of deriving importance sampling densities for numerous components in large-scale systems. Here, we propose a graph learning approach, called importance sampling based on graph autoencoder (GAE-IS), to integrate a modified graph autoencoder model, termed a criticality assessor, with the cross-entropy-based importance sampling method. GAE-IS effectively decouples the criticality of components from their vulnerability to disastrous events in the workflow, demonstrating notable transferability and leading to significantly reduced computational costs of importance sampling in large-scale networks. The proposed methodology improves sampling efficiency by one to two orders of magnitude across several road networks and provides more accurate probability estimations.

## INTRODUCTION

The vulnerability of engineered systems to extreme events poses significant risks, including substantial loss of life, economic damage, and threats to national security.[1,2] These extreme events range from catastrophic disasters that can devastate infrastructure such as power grids and transportation networks, leading to the failure of critical lifelines,[3,4] to accidents caused by aging components or system malfunctions[5,6] and severe cascading failures triggered by load fluctuations.[7,8] Although these extreme events have a low probability of occurrence, residing in the tail of the probability distribution, their social and economic impacts are often immense and unacceptable. Therefore, assessing the reliability of engineering systems under such extreme conditions, known as "corner cases," is essential. This assessment serves as the cornerstone for reliable, robust, and resilient system planning and design,[9,10] identifying potential risks and vulnerabilities[11] and developing effective countermeasures[12,13] to mitigate the likelihood or impact of such events. However, given the rarity of extreme events and the challenges in characterizing them,[14] achieving computational efficiency in reliability or resilience assessments becomes a significant challenge, particularly when using traditional methods such as the Monte Carlo simulation.[15,16] For instance, estimating the probability of extreme failure scenarios at a level of $10^{-6}$ with a standard error of 10% would require sample sizes on the order of $10^8$. In practice, simulating the performance of complex nonlinear engineering systems under numerous failure scenarios is computationally intensive, presenting significant obstacles to accurately evaluating system reliability and resilience under extreme conditions.


This is an open access article under the CC BY license (http://creativecommons.org/licenses/by/4.0/).

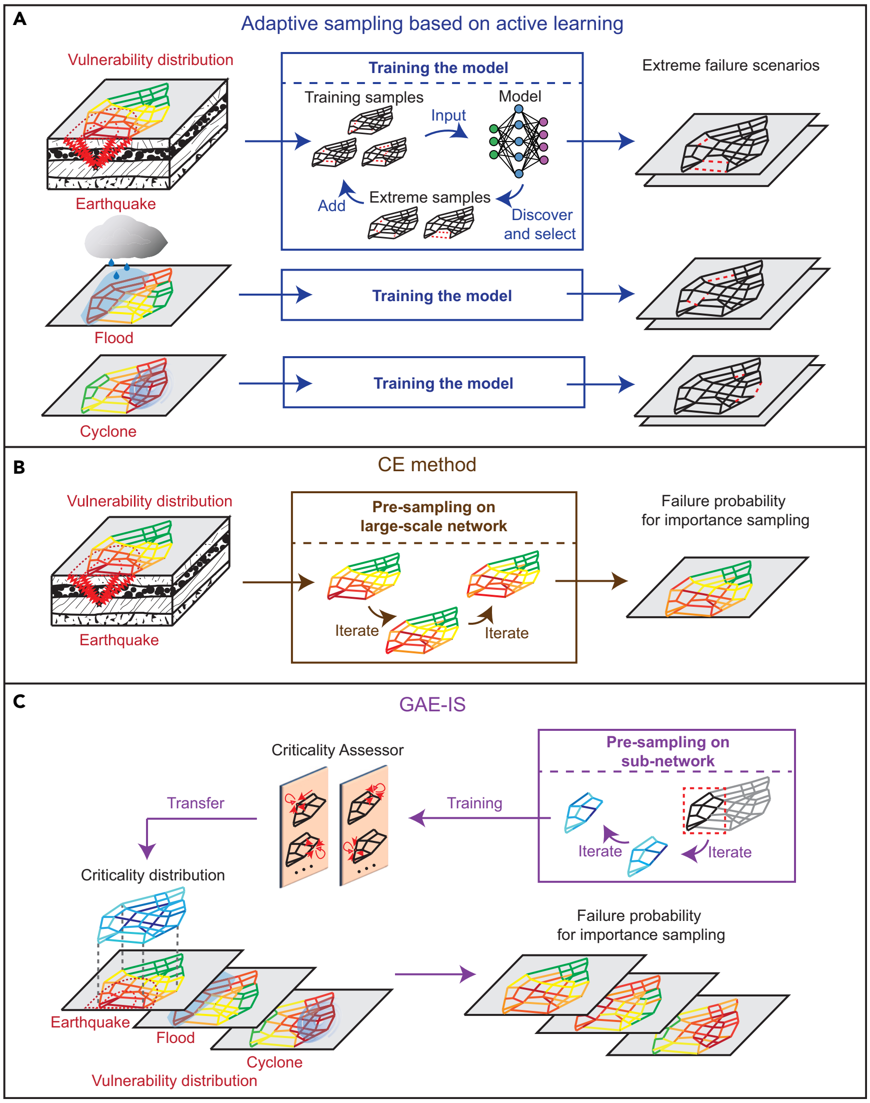


**Figure 1. Comparison of GAE-IS (importance sampling based on graph autoencoder) with the existing CE (cross-entropy) method and adaptive sampling method on a road network for estimating the probability of extreme failures under various disaster scenarios**

A method that has gained prominence in recent research for rare event analysis is adaptive sampling based on active learning. This approach actively chooses samples by optimizing a predefined acquisition function,[17] which accelerates the convergence of extreme scenario probability estimates. This method has been applied in the fields of safety and reliability analysis, such as accident rate detection for autonomous vehicles,[18] reliability assessment of engineering structures,[19,20] and the discovery and prediction of rogue ocean waves.[14] However, this method is data driven and relies on training samples. When evaluating the probability of extreme failures for infrastructure systems under disastrous events, it is necessary to retrain the model whenever there is a change in the type of disaster or in the spatial distribution of the disaster's intensity.

Another commonly used method is importance sampling,[21,22] which has been recognized as an effective method for reducing variance in the statistical analysis of extreme events. This method employs an auxiliary distribution known as the importance sampling density (ISD). By transforming samples of interest, which are unlikely to be obtained from the original distribution, into samples with higher probability density under the ISD, importance sampling significantly improves sampling efficiency related to rare events. This technique has found extensive application in risk and reliability assessments across various domains, such as credit risk evaluation in portfolio investments in the financial sector,[23,24] multiscenario driving safety testing for autonomous vehicles,[25,26] and vulnerability and reliability assessment for power systems,[27,28] as well as other engineered systems characterized by series-parallel structures and complex failure patterns.[29,30] These applications highlight the versatility and effectiveness of importance sampling in addressing challenges associated with rare and extreme events.

However, a primary challenge in implementing importance sampling lies in identifying an appropriate ISD, as it significantly affects both the accuracy of probability estimation and the degree of sampling efficiency improvement.[15,22] Various methods have been employed to approximate the ISD, including parametric density approximation methods such as Gaussian mixture models[31,32] and nonparametric methods like adaptive kernel density estimators.[33,34] Among these, the cross-entropy (CE) method is a widely used adaptive sampling technique. It approximates the ISD by minimizing the Kullback-Leibler (KL) divergence between the theoretically optimal ISD and a chosen parametric family of distributions.[35] Despite its effectiveness, the CE method and similar approaches face limitations when dealing with high-dimensional problems.[36] As the dimensionality of the variables increases, the number of unknown model parameters and required sample size escalate rapidly.[37] This poses significant challenges when applying these methods to estimate extreme failure probabilities in large-scale networks with numerous components, where the complexity and computational costs can become prohibitive. For infrastructure systems such as transportation networks, which are often large-scale and complex, resilience assessment and optimization in the existing efforts typically measure resilience using the overall system performance under a specific extreme event[38] or the mean of system performances under a group

CellPress
OPEN ACCESS

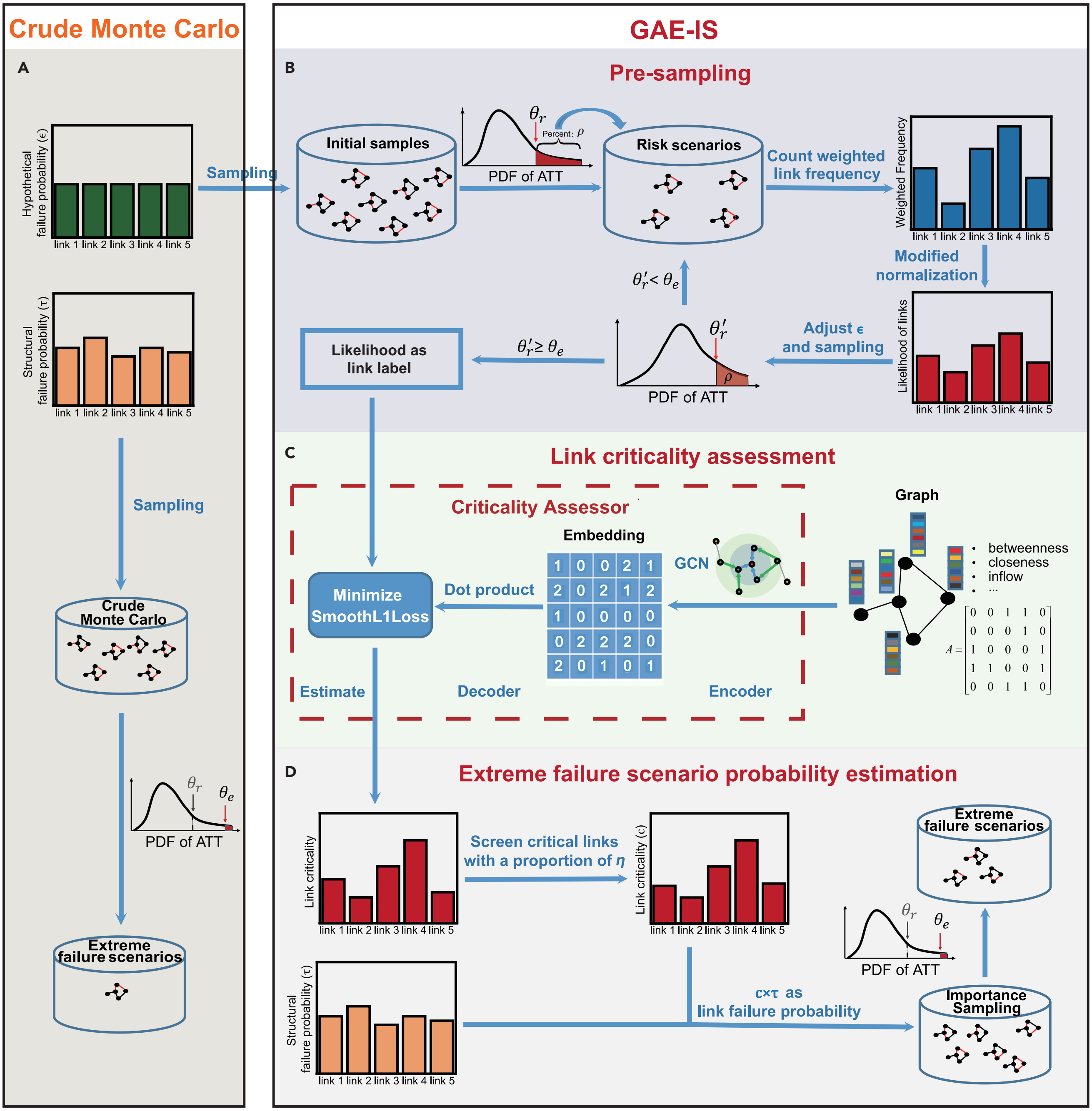


**Figure 2. Overview of the GAE-IS workflow**

This figure illustrates the process for sampling extreme failure scenarios that lead to significant degradation in traffic efficiency due to link failures in a road network.

(A) The crude Monte Carlo method used for presampling and serving as a baseline for sampling extreme failure scenarios.

(B–D) GAE-IS comprises three key components: (B) presampling on a sub-network using the CE method to estimate the likelihood of each link being part of failed link sets in risk scenarios, (C) assessing link criticality using the criticality assessor, and (D) sampling extreme failure scenarios based on the link criticality provided by the criticality assessor.

of failure scenarios.[39] Addressing the challenge of evaluating the probability of extreme failures will enable a more comprehensive resilience assessment from the perspective of extreme value statistics.[40,41]

In this study, we propose a graph learning approach named importance sampling based on graph autoencoder (GAE-IS) to efficiently sample extreme failure scenarios and estimate their probabilities in infrastructure networks, taking transportation

**Table 1. Node features of the road network**

| Feature category | Feature | Description | Feature function |
|---|---|---|---|
| Topological centrality metrics | in-degree | number of incoming edges | reflects a node's connectivity |
| | out-degree | number of outgoing edges | |
| | betweenness centrality | number of shortest paths through a node | reflects the node's role in efficiently connecting nodes |
| | closeness centrality | the mean distance from the node to all other nodes in the network | reflects the position of a node in the network |
| | eigenvector centrality | determined by the centrality of the node's neighbors | reflects the importance of a node's neighbors |
| Traffic flow attributes | inflow | flow into the node | reflects the node's load |
| | outflow | flow out of the node | |
| | a binary indicator for whether the node serves as an origin or a destination point for travel demands | 1 for yes, 0 for no | reflects the role of the node in travel demand distribution |
| | the remaining capacity of a node as a starting point | difference between the sum of traffic capacity of all links starting at the node and the sum of traffic flow on links starting at the node | reflects the redundancy in capacity of roads connected to the node |
| | the remaining capacity of a node as an ending point | difference between the sum of traffic capacity of all links ending at the node and the sum of traffic flow on links ending at the node | |
| | the free-flow speed of a node as a starting point | sum of the free-flow speeds of all links starting at the node | reflects the hierarchy of roads connected to the node; higher-hierarchy roads are typically designed to have higher free-flow speeds |
| | The free-flow speed of a node as an ending point | sum of the free-flow speeds of all links ending at the node | |

networks as an example. The experiments on road networks demonstrate the effectiveness of the proposed methodology, showcasing its ability to offer a computationally feasible approach for assessing the resilience of large-scale infrastructure networks.

## Overview of the GAE-IS approach

GAE-IS represents an advancement over the traditional CE method and addresses the challenge of sampling difficulties associated with obtaining the ISD function in large-scale networks with numerous components. The proposed GAE-IS method offers two key advantages, as illustrated in Figure 1.

(1) Transferability of the proposed criticality assessor via a graph autoencoder (GAE): we develop a graph learning model based on a GAE, referred to as the criticality assessor, to assess the criticality of network components on overall network functionality. In this study, the criticality of a network component is defined as the extent to which the component's failure results in the degradation of network performance, taking multiple components' simultaneous failure into consideration. The criticality assessor, trained on a small-scale subnetwork, can be effectively transferred to the large-scale network it belongs to, thereby facilitating the efficient determination of the ISD function without requiring extensive presampling on the larger target network as shown in Figure 1B. Additionally, due to its transferability, the number of parameters in the model remains constant regardless of the scale of the system or the dimensionality of the variables. This effectively addresses the challenges of parameter and sample-size explosion typically faced by existing importance sampling methods, such as the CE method, in high-dimensional systems.

(2) Decoupling criticality from physical failure characteristics in the workflow: we assume that vulnerability is related to the spatial distribution of the disruptive intensity caused by potential hazards. Combined with the structural fragility of components, the resulting risk of component failure is referred to as the vulnerability distribution, as shown in Figure 1A. The criticality of components is independent of the vulnerability distribution. By integrating the spatial distribution of criticality, termed as the criticality distribution, as shown in Figure 1C, with the network's vulnerability distribution in the context of a catastrophic event, we can derive ISD functions of components for GAE-IS. This approach eliminates the need to retrain the model across various types of disasters or various vulnerability distributions, which are inevitable in the adaptive sampling methods shown in Figure 1A.

GAE-IS is designed to accommodate a range of component failure patterns, including various levels of capacity degradation, and is capable of handling both homogeneous and

CellPress
OPEN ACCESS

**Table 2. Experimental network settings**

| Network | Berlin: Berlin-Friedrichshain (for training) | Berlin: BMPFC | Chicago: Partial CS (for training) | Chicago: CS |
|---|---|---|---|---|
| Number of nodes | 224 | 975 | 219 | 933 |
| Original number of links | 523 | 2,184 | 638 | 2,950 |
| Number of links after removing extra connector links | 387 | 1,607 | 638 | 2,950 |
| Number of zones | 23 | 98 | 77 | 387 |
| Number of OD pairs | 506 | 9,505 | 5,929 | 142,890 |
| Number of trips | 11,205 | 23,648 | 362,858 | 1,260,907 |
| Initial ATT | 82.30 (s) | 117.81 (s) | 9.83 (min) | 14.43 (min) |
| Initial c-link capacity (veh/h) | 999,999 | 999,999 | 49,500 | 49,500 |
| Modified c-link capacity (veh/h) | 7,000 | 7,000 | 25,000 | 25,000 |

| Network | Anaheim: Partial Anaheim (for training) | Anaheim: Anaheim | Northern part of Gold Coast: Partial NGC (for training) | Northern part of Gold Coast: NGC |
|---|---|---|---|---|
| Number of nodes | 138 | 416 | 281 | 1,051 |
| Number of links | 294 | 914 | 592 | 2,417 |
| Number of zones | 15 | 38 | 67 | 249 |
| Number of OD pairs | 210 | 1,406 | 4,489 | 62,001 |
| Number of trips | 12,166 | 104,694 | 6,375 | 23,443 |
| Initial ATT | 10.87 (min) | 12.87 (min) | 4.77 (min) | 7.13 (min) |

heterogeneous failure probabilities. Moreover, it takes into account both the network's topology and its spatial travel demand distribution characteristics. As a result, although focusing on transportation systems in this study, the proposed methodology has the potential to be applied to other infrastructure systems characterized by network topological features and cyber or physical flows transferring in it, such as water distribution, power, and communication systems.

## METHODS

### The workflow of GAE-IS

Road transportation networks are represented as directed graphs consisting of nodes and links. Nodes represent intersections and origin-destination (OD) points where travel demand originates or terminates, while links represent road segments. The average travel time (ATT) is used as the system performance indicator to evaluate the post-event system level of service. It measures the extent of functionality loss in the system, thereby aiding in determining whether a failure scenario should be classified as an extreme failure scenario. The ATT is calculated as:

$$\mathrm{ATT} = \frac{1}{D}\sum_{i} t_{l_i} Q_{l_i}, \qquad \text{(Equation 1)}$$

where $D$ represents the total travel demand in the network, $Q_{l_i}$ is the traffic volume on link $l_i$, and $t_{l_i}$ represents the travel time on link $l_i$, which is calculated using the Bureau of Public Roads (BPR) function:

$$t_{l_i} = t_{l_i 0} \times \left(1 + \alpha \times \left(\frac{Q_{l_i}}{CA_{l_i}}\right)^{\beta}\right), \qquad \text{(Equation 2)}$$

where $t_{l_i 0}$ is the free-flow travel time of link $l_i$ and $CA_{l_i}$ is the link capacity; $t_{l_i 0}$ and $CA_{l_i}$ are predetermined by the network configuration given in the transportation network dataset,[42] and parameters $\alpha$ and $\beta$ take 0.15 and 4, respectively, referring to the literature.[43]

In our study, $Q_{l_i}$ is the output of the traffic assignment model, i.e., user equilibrium (UE)[44]; the formulation of UE can be found in Appendix A in the supplemental methods. UE-based traffic assignment is affected by various factors, including network topology, capacity configurations, spatial distribution of travel demand, and the adaptability of network users to congested traffic conditions. It is a complex nonlinear and self-organizing process, with computational complexity increasing significantly with the size of the network.[45] This study focuses on failure scenarios with capacity degradations of road segments. Therefore, for each failure scenario, the travel demand is reassigned, given the decreased capacity of affected links to determine the volume $Q_{l_i}$ on each link. For the failed links, the parameter $CA_{l_i}$ in the BPR function is also reduced accordingly. In future work, scenarios with complete link interruption can be studied by reassigning the remaining connected travel demand and imposing a penalty on the disconnected travel demand to calculate the ATT.

Given factors such as geographic location and structural characteristics of road segments, there exists a spatial heterogeneous failure probability $\tau$ for each link. We assume that the structural failure of links is mutually independent. Failure scenarios refer to various combinations of failed links within the network, and extreme failure scenarios are defined as those with ATT exceeding the threshold $\theta_e$. To set the threshold for extreme failure scenarios, follow these steps. First, sample $5\times10^3$ failure scenarios using a crude Monte Carlo method. Then, fit the right-skewed ATT with a gamma distribution or use kernel density estimators to account for multimodal ATT

**Table 3. Experimental parameter settings**

| Parameters/hyperparameters | | BMPFC | Anaheim | NGC | CS |
|---|---|---|---|---|---|
| GAE-IS | $\tau$ | 0.1 | 0.1 | 0.05 | seismic-based (mean: 0.0156) |
| | $\epsilon$ | 0.1 | | | |
| | $N_t$ | $1 \times 10^4$ | | | |
| | $\rho$ | 10% | | | |
| | $\eta$ | 0.15 | | | |
| | capacity decrease | 50% | | | |
| Criticality assessors | output channels of conv layers | 40, 20 | | | |
| | Output channels of FC layers | 20 | | | |
| | activation function for each layer | ReLU | | | |
| | loss function | SmoothL1Loss | | | |
| | optimizer | Adam | | | |
| | learning rate | 0.002 | | | |
| | weight decay | 0.0001 | | | |
| | epoch | 300 | 200 | 100 | 200 |

distributions. The 99.75th and 99.95th percentiles of the ATT distribution are considered as extreme failure thresholds. In practice, the threshold can be adjusted flexibly to meet the specific requirements of the application scenario, and the fitting methods can be further adjusted according to the specific probability distribution characteristics of ATT in the sampled scenarios. Although this provides a reference setting for $\theta_e$, fitting the ATT distribution introduces errors, making percentile-based probability estimates inaccurate. Accurate probabilities of extreme failure scenarios still require estimation through the proposed GAE-IS method. Overall, in the context of this study, extreme failure scenarios in the road network are those in which multiple road segments fail simultaneously, resulting in a significant decline in traffic efficiency, i.e., significant increase in ATT.

The criticality of links is unrelated to their structural condition and the spatial distribution of the disruptive intensity. Therefore, we assume that links in the sub-network (training network) have a homogeneous hypothetical failure probability $\epsilon$ for the training of the criticality assessor. This allows us to assess the criticality of links and identify those that are critical for network functionality, given the same failure probability among links. By increasing the failure probabilities of these critical links, we have a greater chance of obtaining more extreme failure scenarios. Unlike studies focusing on the impact of individual component failures on system functionality,[46,47] our study emphasizes the criticality of links under simultaneous multiple component failures.

The crude Monte Carlo is employed to randomly sample $N_t$ network failure scenarios with a given link failure probability, $\epsilon$, in the training network, creating the initial sample set (Figure 2A). A predetermined percentage (i.e., the $\rho$ percentage) of these samples, which show substantial degradation in network performance, is selected as risk scenarios. The threshold for ATT corresponding to these risk scenarios is denoted as $\theta_r$. The likelihood of each link appearing in the set of failed links within these risk scenarios is calculated, where a higher likelihood indicates a more critical link. This likelihood is utilized to adjust the hypothetical failure probability of the link to increase it for more critical links and decrease it for less critical links. Then, another set of $N_t$ samples is generated, and the risk scenarios are updated with a new risk scenario threshold, $\theta'_r$. This iterative process continues until the risk scenario threshold $\theta'_r$, associated with the $\rho$ percentage of the samples, exceeds $\theta_e$ (for details of the iterative process refer to Appendix B in supplemental methods). This iterative procedure is referred to as presampling, as shown in Figure 2B.

Next, the criticality assessor uses inputs such as the road network's adjacency matrix and node attributes related to multiple topological centrality metrics and traffic flow (details in the next sub-section) to learn the feature representation of each link's impact on network performance degradation, as shown in Figure 2C. It estimates and outputs the likelihood of each link being part of the set of failed links in risk scenarios, thereby quantifying the criticality of the link. The estimated criticality is then used as a coefficient to adjust links' structural failure probabilities, as shown in Figure 2D. Links are sorted by their criticality in descending order. The top $\eta$ proportion of links is considered critical, while the bottom $\eta$ proportion is deemed noncritical, leading to adjustments in their failure probabilities. For details on how to modify the estimated criticality based on parameter $\eta$, please refer to Appendix B in the supplemental methods. This approach integrates link criticality with failure risk to derive the ISD function for each link, which is associated with the adjusted failure probability of that link. Ultimately, the ISD function for failure scenarios is determined by the ISD function of each link. When the structural failure probability of a link changes, there is no need to reassess the criticality. Thus, the GAE-IS can be applied to networks considering various types of disasters and spatial vulnerability distributions, enhancing computational efficiency in assessing system resilience across different vulnerability characteristics.

It's worth mentioning that transferring a trained criticality assessor to another network requires providing only the adjacency matrix and node attributes of that network to obtain the new criticality of links, without the need of presampling.

## The criticality assessor for obtaining the ISD function

The criticality assessor is designed to assess link criticality and hence to obtain the ISD function for sampling extreme failure scenarios. This model is based on the GAE framework, an unsupervised deep learning model proposed by Kipf and Welling.[48]

CellPress
OPEN ACCESS

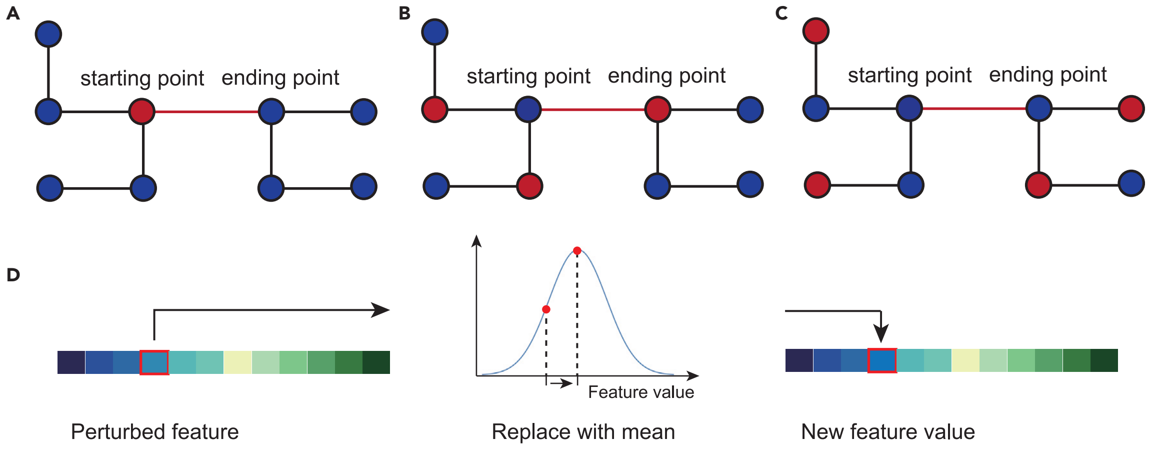


**Figure 3. Schematic diagram of the feature perturbation experiment**

(A–C) The diagrams illustrate the feature perturbation at: (A) the starting point, (B) the one-hop neighbors of the starting point, and (C) the two-hop neighbors of the starting point.

(D) The perturbation method involves replacing the specified feature dimension of the perturbed node with the mean value of that feature dimension across all nodes in the network. The perturbation method for the ending point and its one-hop and two-hop neighbors is the same as that for the starting point.

The GAE entails utilizing the known adjacency matrix of an incomplete graph and the feature matrix of nodes as inputs, applying graph convolution to encode and learn node representations and then reconstructing the original network using a decoder. The model parameters are optimized by minimizing the graph reconstruction error to address link prediction tasks.

In the proposed criticality assessor, the node feature matrix consists of both topological metrics and traffic flow attributes. Table 1 provides a detailed description of these features. Each node is represented by a 12-dimensional feature vector. During the data preprocessing stage, each feature dimension of the node is standardized. To encode the input data, we use two layers of graph convolutional networks followed by two fully connected layers, with link capacity serving as the edge attribute. Rather than tackling link prediction tasks with a traditional GAE model, the proposed criticality assessor employs the inner product of node embedding vectors in its decoding module to estimate the likelihood of link occurrence within a set of failed links in risk scenarios.

In transportation networks, traffic flow can vary significantly across different directions of the same road segment due to heterogeneous spatial travel demand distributions. Consequently, the impact of a road segment's failure on network performance may differ depending on its direction. To account for this, this study models the road transportation network as a directed graph. However, the traditional GAE was designed for undirected graphs. For example, using the inner product of endpoint embedding vectors to calculate the probability of a link's existence does not differentiate between directed links from node $i$ to node $j$ and those from node $j$ to node $i$. To address this issue, previous studies have developed node embedding methods[49] or decoding techniques[50] specifically for directed graphs. We adopt the approach proposed by Ou et al.,[51] which involves training embedding vectors separately for the starting and ending points of links, thereby allowing us to differentiate bidirectional links.

To train the criticality assessor, we assign a real-valued label to each link in the network. Details of the label generation process are provided in Appendix B in the supplemental methods.

The parameter optimization for the criticality assessor is achieved by minimizing the error between the estimated likelihood of links (calculated as the inner product of node embedding vectors) and their corresponding real-valued labels. The specific hyperparameter settings of the model are presented under "experimental setup."

## Importance sampling theory

Importance sampling is a technique that enhances the number of specific samples by generating samples from an introduced auxiliary distribution.[15] We consider the objective of estimating the value of $E(f(x)) = \int_{\mathcal{D}} f(x)g(x)dx$, where $g(x)$ is a probability density function defined on $\mathcal{D} \subseteq \mathbb{R}^d$, $g$ is referred to as the nominal distribution, and $f$ represents the integrand. For all $x \notin \mathcal{D}$, $g(x) = 0$. If $q(x)$ is a positive probability density function on $\mathbb{R}^d$, then:

$$E(f(x)) = \int_{\mathcal{D}} f(x)g(x)dx = \int_{\mathcal{D}} \frac{f(x)g(x)}{q(x)} q(x)dx = E_q\left(\frac{f(X)g(X)}{q(X)}\right), \quad \text{(Equation 3)}$$

where $E_q(\cdot)$ represents expectation for $X \sim q$, $w = g(x)/q(x)$ is the importance weight, $q(x)$ represents the ISD function, and $q$ is referred to as the importance distribution.

By sampling $n$ instances from $q$, the expectation can be estimated using the sample mean:

$$\widehat{E}(f(x)) = \frac{1}{n}\sum_{i=1}^{n} \frac{f(X_i)g(X_i)}{q(X_i)}, \quad X_i \sim q. \quad \text{(Equation 4)}$$

Each instance from $q$ is weighted by the importance weight to keep the unbiasedness. It is important to note that, in the process of substituting $q$ for $g$ during the sampling, a vital requirement is that $q(x) > 0$ whenever $f(x)g(x) \neq 0$.

Then, the variance of probability estimation (VPE) is given by:

$$\widehat{\sigma}_q^2 = \frac{1}{n}\sum_{i=1}^{n}\left(\frac{f(X_i)g(X_i)}{q(X_i)} - \widehat{E}(f(x))\right)^2, \quad \text{(Equation 5)}$$

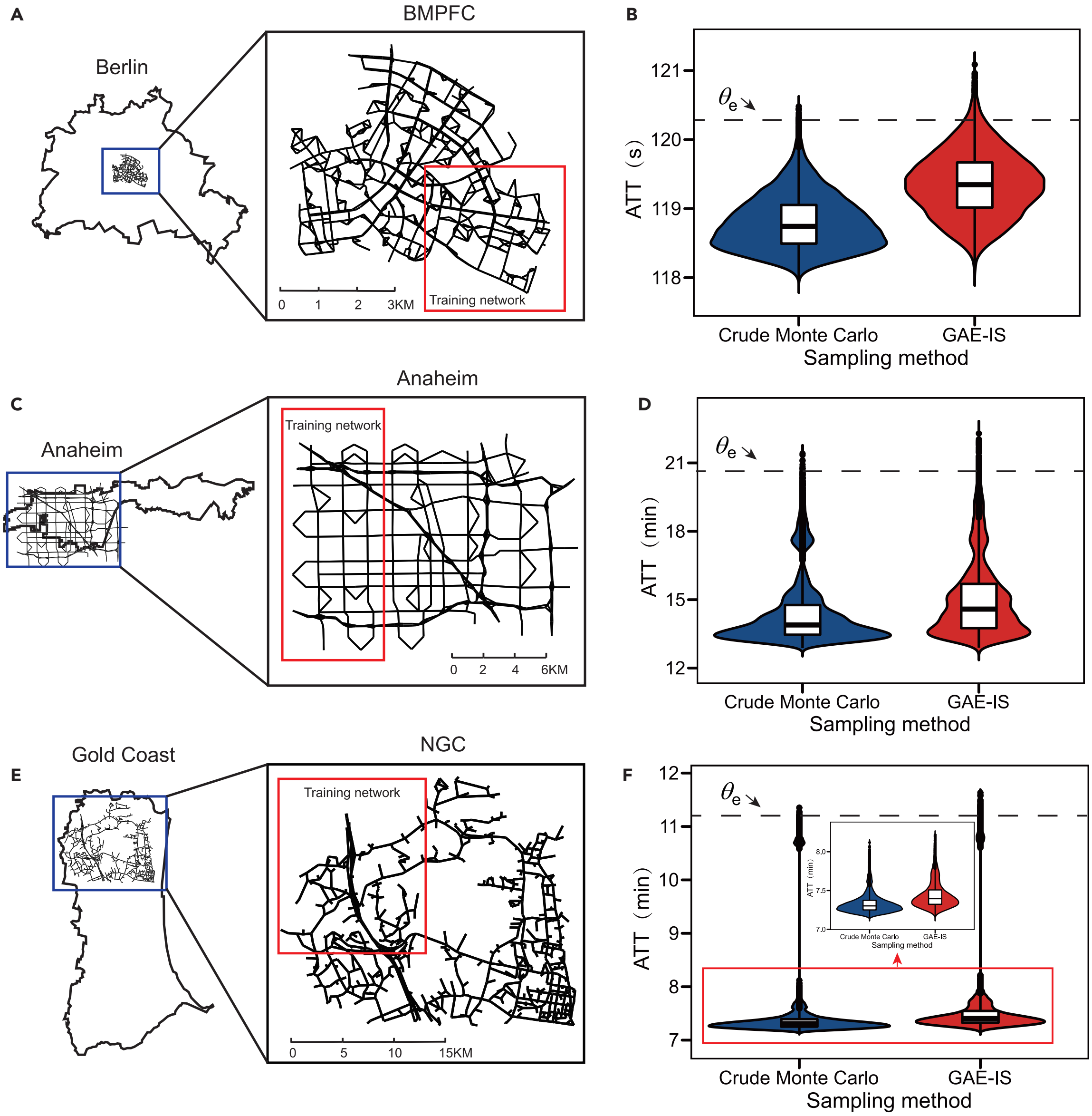


**Figure 4. Sampling results for road networks in BMPFC, Anaheim, and NGC**
(A) Schematic diagram of Berlin's city boundaries and the BMPFC network.
(B) Violin plot of the ATT distribution for $2\times10^4$ failure scenarios sampled from the BMPFC network.
(C) Schematic diagram of Anaheim's city boundaries and the Anaheim network.
(D) ATT distribution for $2\times10^4$ failure scenarios sampled from the Anaheim network.
(E) Schematic diagram of Gold Coast's city boundaries and the NGC network.
(F) ATT distribution for $2\times10^4$ failure scenarios sampled from the NGC network.

where a high VPE indicates greater deviation of data points from the mean, resulting in increased instability in the estimated probability.

## The proposed GAE-IS method

The proposed GAE-IS method focuses on estimating the probability of extreme failure scenarios involving the failure of multiple link combinations in a transportation network. Each failed link may experience a partial decrease in its capacity, leading to an increase in the ATT within the network. If the ATT of a specific failure scenario exceeds the threshold $\theta_e$, this scenario is classified as an extreme failure scenario:

$$f(x) = \mathbb{I}_{\boldsymbol{x}}\{ATT > \theta_e\} = \begin{cases} 1 \; ATT > \theta_e \\ 0 \; ATT \le \theta_e \end{cases}, \qquad \text{(Equation 6)}$$

where $\boldsymbol{x}$ denotes the variables of the link states in a specific failure scenario, $\boldsymbol{x} = (s_{l_1}, s_{l_2}, \ldots, s_{l_N})$, $s_{l_i}$ represents the state of link $l_i$, with a value of 1 indicating failure and a value of 0 indicating

**Table 4. Sampling results of GAE-IS in case studies**

| Network | Presampling time (hour) | | Sample size | $\theta_e$ | $\hat{p}$ | VPE | $R$ |
|---|---|---|---|---|---|---|---|
| | Sub-network | Large-scale network | | | | | |
| BMPFC | 3.92 | 46.00 | $2 \times 10^4$ | 120.30 | $7.40 \times 10^{-4}$ | $9.91 \times 10^{-4}$ | 1 |
| | | | | 120.75 | $1.44 \times 10^{-6}$ | $1.76 \times 10^{-8}$ | 82 |
| Anaheim | 1.98 | 18.67 | $2 \times 10^4$ | 20.47 | $5.84 \times 10^{-4}$ | $9.13 \times 10^{-4}$ | 1 |
| | | | | 21.89 | $2.88 \times 10^{-6}$ | $6.40 \times 10^{-8}$ | 45 |
| NGC | 6.64 | 125.00 | $2 \times 10^4$ | 11.28 | $5.12 \times 10^{-5}$ | $9.48 \times 10^{-6}$ | 5 |
| | | | | 11.58 | $4.60 \times 10^{-7}$ | $3.31 \times 10^{-9}$ | 139 |

normal operation, and $N$ represents the total number of links. In a failure scenario where the link state is $\boldsymbol{x}$, if the ATT exceeds the threshold $\theta_e$, the indicator function, $I_{\boldsymbol{x}}\{ATT > \theta_e\}$, takes the value of 1; otherwise, it is 0.

The objective is to estimate the value of $P(ATT > \theta_e)$, denoted as $p$, which represents the probability of extreme failure scenarios in the network. The probability $p$ can be calculated as follows:

$$p = \sum_{\boldsymbol{x} \in \boldsymbol{X}} I_{\boldsymbol{x}}\{ATT > \theta_e\} g(\boldsymbol{x}), \tag{Equation 7}$$

where $\boldsymbol{X}$ represents the feasible domain of $\boldsymbol{x}$, encompassing all possible link state vectors. The term $g(\boldsymbol{x})$ represents the probability density function of failure scenarios.

For a network with $N$ links, each of which has a mutually independent structural failure probability $\tau$ $(0 < \tau \leq 1)$, the probability density function $g(\boldsymbol{x})$ of a failure scenario can be calculated as follows, where $z(s)$ represents the probability density function of the link state:

$$g(\boldsymbol{x}) = \prod_{i=1}^{N} z(s_{l_i}), \forall \boldsymbol{x} \in \boldsymbol{X}, \tag{Equation 8}$$

$$z(s_{l_i}) = s_{l_i}\tau_{l_i} + (1 - s_{l_i})(1 - \tau_{l_i}). \tag{Equation 9}$$

Due to the impracticality of exhaustively enumerating all possible failure scenarios, the probability $p$ is estimated through sampling techniques. In the proposed methodology, we utilize the criticality assessor to estimate the likelihood of link $l_i$ appearing in the set of failed links in risk scenarios, as a measure of link criticality. The modified criticality is denoted as $c_{l_i}$ $(c_{l_i} > 0)$. We take $c_{l_i}$ as the adjustment coefficient for the structural failure probability of the link in the ISD function to sample failure scenarios using GAE-IS. Then, the ISD function $q(\boldsymbol{x})$ for a failure scenario is calculated as follows, where $d(s)$ represents the ISD function of the link state:

$$q(\boldsymbol{x}) = \prod_{i=1}^{N} d(s_{l_i}), \forall \boldsymbol{x} \in \boldsymbol{X}, \tag{Equation 10}$$

$$d(s_{l_i}) = s_{l_i}\varphi(c_{l_i}\tau_{l_i}) + (1 - s_{l_i})\left(1 - c_{l_i}\tau_{l_i}\right), \tag{Equation 11}$$

$$\varphi(c_{l_i}\tau_{l_i}) = \min(c_{l_i}\tau_{l_i}, 1). \tag{Equation 12}$$

By sampling $n$ instances from the distribution $q$ and substituting Equation 6, Equation 8, and Equation 10 into Equation 4, an estimation of the probability $p$ under $q(\boldsymbol{x})$ is given by:

$$\hat{p}_q = \frac{1}{n}\sum_{i=1}^{n} \frac{I_{\boldsymbol{x}_i}\{ATT > \theta_e\} g(\boldsymbol{x}_i)}{q(\boldsymbol{x}_i)}, \boldsymbol{x}_i \sim q. \tag{Equation 13}$$

To validate the sampling efficiency of the proposed GAE-IS method, we measure the reduction in VPE achieved by GAE-IS compared to crude Monte Carlo, with the same sample size. In the case of crude Monte Carlo, where samples follow a Bernoulli distribution, the VPE can be calculated as $p(1 - p)$. Then, we can obtain the variance reduction ratio as follows:

$$R = \frac{\hat{p}_q(1 - \hat{p}_q)}{\hat{\sigma}_q^2} = \frac{\hat{p}_q(1 - \hat{p}_q)/n}{\mathrm{se}(\hat{p}_q)^2}, \tag{Equation 14}$$

where $\hat{\sigma}_q^2$ is the VPE in the GAE-IS method and $\mathrm{se}(\hat{p}_q)^2$ is the sampling variance of $\hat{p}_q$, with the sampling variance calculated as follows:

$$\mathrm{se}(\hat{p}_q)^2 = \frac{\hat{\sigma}_q^2}{n} = \frac{1}{n^2}\sum_{i=1}^{n}\left(\frac{I_{\boldsymbol{x}_i}\{ATT > \theta_e\} g(\boldsymbol{x}_i)}{q(\boldsymbol{x}_i)} - \hat{p}_q\right)^2. \tag{Equation 15}$$

In other words, GAE-IS achieves an efficiency improvement by a factor of $R$ compared to crude Monte Carlo within the given sample size.

## Case study networks and data preparation

The performance of the proposed methodology is demonstrated on several real road transportation networks: Berlin-Mitte-Prenzlauerberg-Friedrichshain-Center (BMPFC) in Germany, the northern part of Gold Coast (NGC) in Australia, and Anaheim and Chicago-Sketch (CS) in the United States. The BMPFC, NGC, and Anaheim networks each cover a portion of the city, whereas the CS network covers the whole city. Network configuration parameters are detailed in Table 2.

In our study, we incorporate the capacity of road segments as the edge attribute. In the original network configurations, there are some spurious road segments, referred to as connector links (c-links), which connect each zone centroid to the surrounding links of that zone. These connector links have disproportionately high capacities and a travel time of zero. To ensure balanced message passing and to prevent the model from overly prioritizing nodes or links with exceptionally high edge attributes, we

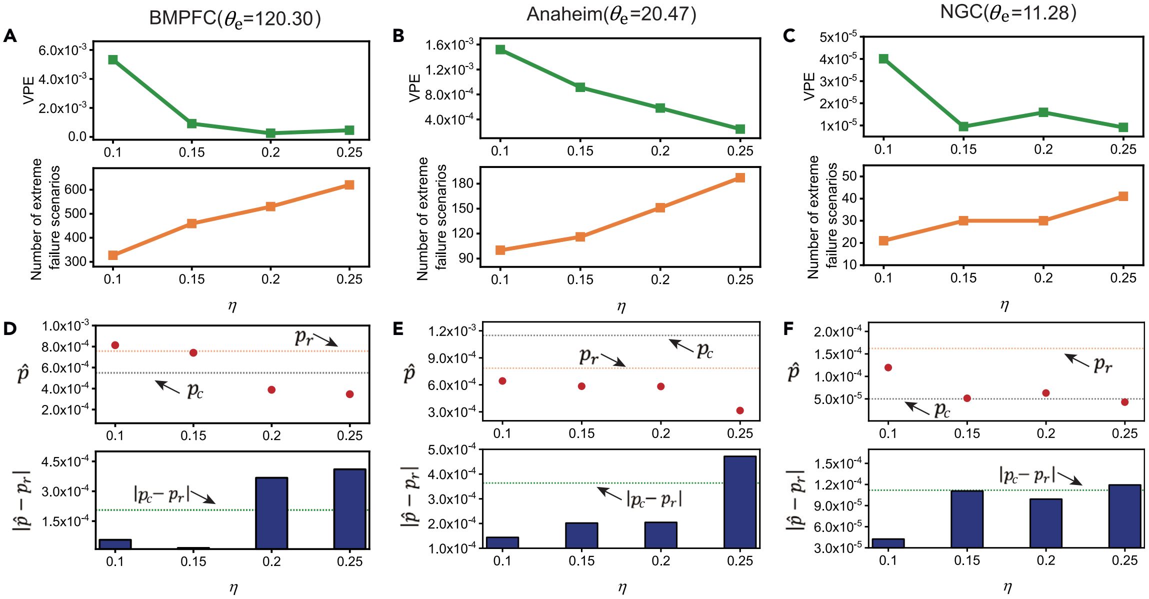


**Figure 5. Sampling results for case networks with four different $\eta$ settings, i.e., 0.1, 0.15, 0.2, and 0.25**

For each parameter setting, $2\times10^4$ failure scenarios were sampled.

(A) Number of extreme failure scenarios sampled and the VPE for the BMPFC network.

(B) Number of extreme failure scenarios sampled and the VPE for the Anaheim network.

(C) Number of extreme failure scenarios sampled and the VPE for the NGC network.

(D) Estimated probabilities of extreme failure scenarios and errors compared to the reference probability for the BMPFC network.

(E) Estimated probabilities of extreme failure scenarios and errors compared to the reference probability for the Anaheim network.

(F) Estimated probabilities of extreme failure scenarios and errors compared to the reference probability for the NGC network.

Red dots represent probabilities estimated via GAE-IS, orange dashed lines denote the reference probability ($p_r$) derived through $5\times10^5$ crude Monte Carlo samples, and gray dashed lines indicate the probabilities (denoted as $p_c$) estimated from $2\times10^4$ crude Monte Carlo samples. Blue bars show the absolute error between the GAE-IS estimates and the reference probability, while green dashed lines represent the absolute error between the probabilities estimated from $2\times10^4$ crude Monte Carlo samples and the reference probability.

adjust the capacity for connector links that have significantly higher capacity compared to regular links. For the BMPFC network, the capacity of the connector links is set to 2.5 times the maximum capacity of regular links. In the CS network, which has a broader range of link capacities, the capacity of connector links is set to 5 times the median capacity of the links. The road networks of Anaheim and NGC do not include connector links, and therefore no preprocessing is required. To conclude, the introduction of scaling factors is intended to address the network configuration deficiencies, which do not affect the performance evaluation of the proposed methodology.

## Experimental setup

The parameter settings of applying GAE-IS for sampling failure scenarios in all networks, along with hyperparameter settings for the criticality assessors, are shown in Table 3. Figure S1 illustrates the loss function values and the corresponding VPE for extreme failure scenarios across different epochs. The cross-referencing between the loss function curve and the VPE curve helps us determine the stopping epochs for the training process, where both the loss function value and the VPE, reflecting the sample variance of GAE-IS, converge.

All experiments were conducted on a computer equipped with 64 GB of RAM and an Intel Core i7-11700K processor (3.6 GHz) and running Ubuntu 18.04.1 LTS.

## Details for feature perturbation experiments

To further investigate the features that significantly contribute to the estimation of criticality of links, we conducted feature perturbation experiments[52] on node features. Since the criticality assessor comprises two graph neural network layers, the results were calculated by two-hop neighbors of the two endpoints of each link. Therefore, for each link, we perturbed the features of the nodes at the link's starting point and its one-hop and two-hop neighbors (as shown in Figure 3) as well as the link's ending point and its one-hop and two-hop neighbors separately. The concatenation of these nodes' feature vectors is termed as the extended node feature vector for perturbation experiments. Specifically, we replaced these features with the mean value of that feature across all nodes. The impact of perturbing each dimension of extended node feature vector on the model's output is quantified by the magnitude of change in the estimated link likelihood, denoted as $\Delta h(EV)$, and calculated as follows:

$$\Delta h_{l_i}\left(EV_{jk}^{l_i}\right) = \frac{\left|\widehat{h}_{l_i}^{p}\left(EV_{jk}^{l_i}\right) - \widehat{h}_{l_i}\right|}{\widehat{h}_{l_i}}, \quad \text{(Equation 16)}$$

where $EV_{jk}^{l_i}$ represents the $k$-th feature of the $j$-th node in the extended node feature vector of link $l_i$, $\widehat{h}_{l_i}$ represents the original estimated likelihood for link $l_i$, and $\widehat{h}_{l_i}^{p}(EV_{jk}^{l_i})$ represents the estimated likelihood for link $l_i$ after perturbing $EV_{jk}^{l_i}$. When a starting

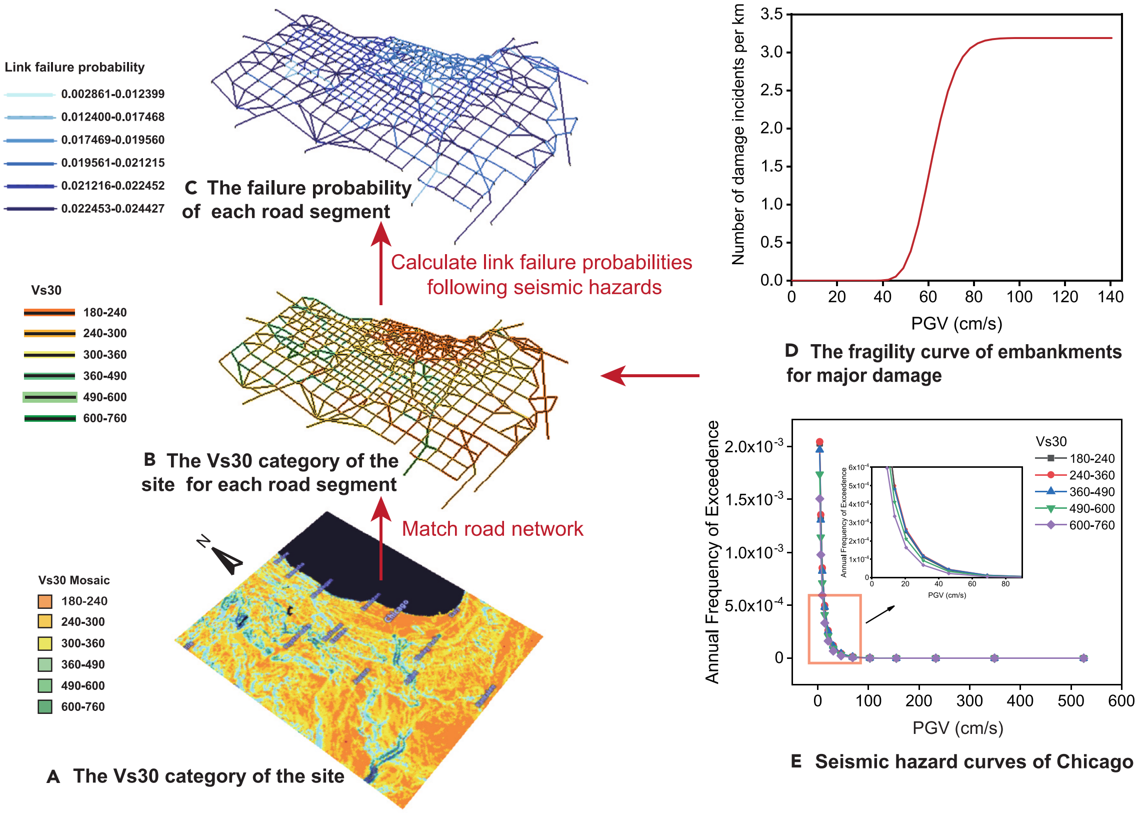


Figure 6. The procedure for estimating link failure probabilities for major damage caused by earthquakes in Chicago

point or ending point has multiple one-hop or two-hop neighbors, the features of each neighbor are perturbed individually, and the mean value of $\Delta h(EV)$ for that range of neighbors is calculated to assess the impact. $\Delta h(EV)$ quantifies the relative impact of feature variations on the model's estimation of link criticality. A larger value of $\Delta h(EV)$ indicates a greater importance of the feature.

It is important to note that, according to the conservation law[53] of traffic flow, when a node serves as an intersection, its inflow is equal to its outflow. As a result, any perturbation in the inflow at an intersection node will correspondingly affect its outflow and vice versa. If a node is an OD point, the perturbations in inflow and outflow are independent of each other. Since the remaining capacity of a node is the difference between its capacity and its flow (as presented in Table 1), when the node's inflow/outflow is perturbed, the remaining capacity of the node as an ending point/starting point will also change accordingly. Similarly, perturbing the remaining capacity will lead to changes in the inflow/outflow.

## RESULTS

### Improved efficiency in sampling extreme failure scenarios using GAE-IS

We evaluated the performance of the proposed GAE-IS method in road transportation systems. The road networks in BMPFC, Anaheim, and NGC are taken as case study networks with homogeneous link failure probabilities. The performance of the GAE-IS under heterogeneous link failure probabilities will be analyzed in a subsequent subsection.

When compared to crude Monte Carlo simulations conducted with the same sample size, GAE-IS demonstrates superior capability in identifying extreme failure scenarios that lead to greater system performance degradation (as shown in Figures 4B, 4D, and 4F). To quantify the reduction in VPE and the sampling efficiency improvement achieved by GAE-IS, the variance reduction ratio $R$ was computed. The case study results show that GAE-IS provides a significant reduction in VPE, indicating enhanced sampling quality compared to crude Monte Carlo. This improvement is particularly pronounced in extreme failure scenarios with higher thresholds $\theta_e$, where sampling efficiency increases by 82, 45, and 139 times for the BMPFC, Anaheim, and NGC networks, respectively (as shown in Table 4). Additionally, the criticality assessor, trained on sub-networks, effectively supports the GAE-IS process when applied to the larger-scale original road networks. On the three case networks, performing presampling on the sub-networks saves 12, 9, and 19 times the computational time, respectively, compared to performing presampling on the original network under the same computational resource setting. These findings demonstrate the transferability of the proposed criticality assessor and highlight the feasibility and effectiveness of using presampling on sub-networks to obtain ISD for large-scale networks at much lower computational cost, taking advantage of the GAE-IS method.

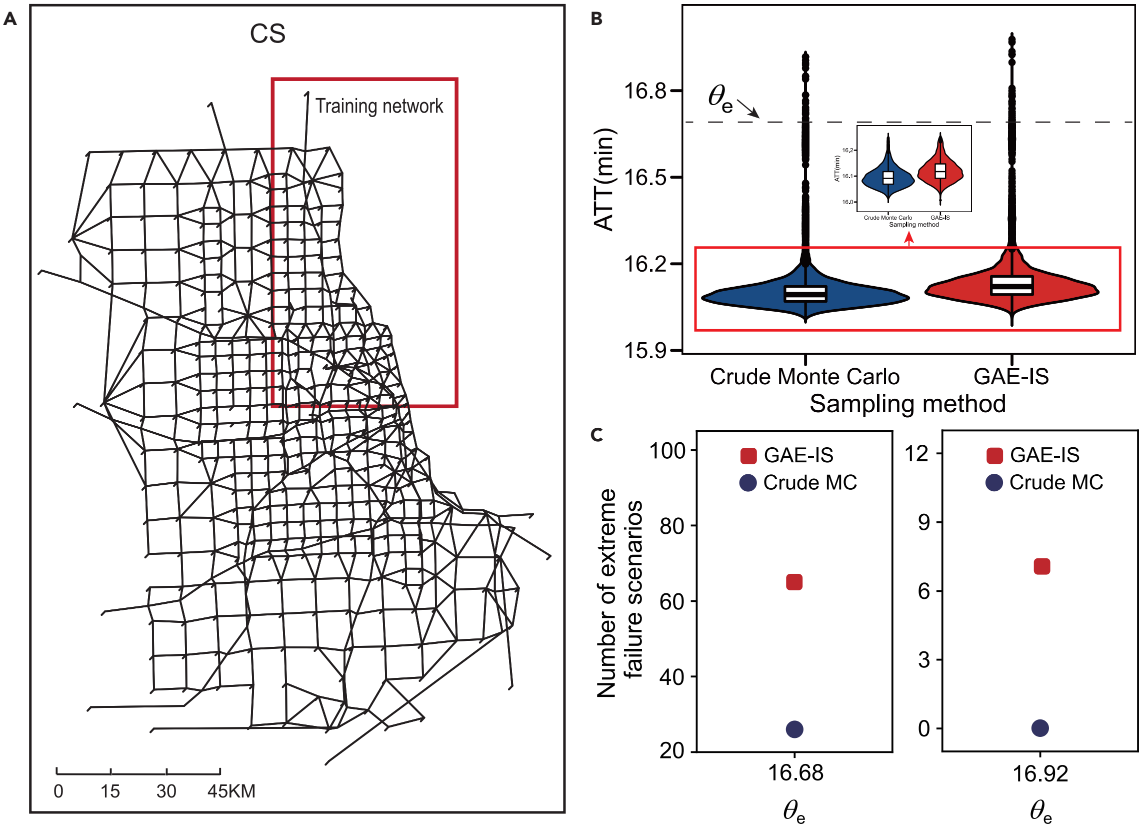


**Figure 7. Sampling results on the Chicago-Sketch road network**

(A) Schematic diagram of the CS network.

(B) The violin plot shows the distribution of ATT for $1\times10^4$ failure scenarios sampled for the CS network.

(C) The number of extreme failure scenarios sampled by GAE-IS and crude Monte Carlo.

## Sensitivity analysis of the GAE-IS performance

The parameter $\eta$ determines how many links in the network are considered critical in importance sampling. Sensitivity analyses are conducted on case networks to assess the impact of different settings of $\eta$ on the performance of GAE-IS.

The efficiency and precision of GAE-IS were evaluated according to two metrics, i.e., the number of sampled extreme failure scenarios and the VPE, respectively. Furthermore, to evaluate the accuracy of probability estimation under limited sample sizes, we compared the probabilities estimated by GAE-IS with the reference probability (denoted as $p_r$) derived from $5\times10^5$ crude Monte Carlo samples. The results, illustrated in Figures 5A–5C, show that increasing $\eta$ enhances the ability of GAE-IS to capture more extreme failure scenarios. This increase in $\eta$ also leads to a reduction in VPE for these scenarios, whereas, when $\eta$ is set to a lower value, the probability estimates provided by GAE-IS align more closely with the reference probability compared to those with larger $\eta$. Moreover, with a lower $\eta$ setting, GAE-IS also outperforms crude Monte Carlo sampling with an equivalent sample size in terms of probability estimation accuracy, as shown in Figure 5. However, as $\eta$ increases, the accuracy of the probability estimates deteriorates, with larger discrepancies observed between GAE-IS and the reference probability $p_r$, as depicted in Figures 5D–5F. This suggests that, at lower values of $\eta$, GAE-IS provides more accurate estimates.

An intriguing observation arises as the parameter $\eta$ increases: despite a reduction in sample variance, there is an increase in the error of probability estimation. This phenomenon indicates that $\eta$ has a significant impact on the accuracy of probability estimates when the sample size is limited. This trade-off highlights the complexity of balancing between sample variance and estimation accuracy in the GAE-IS method. As the number of links with adjusted failure probabilities increases, the variance in the importance weights for failure scenarios also grows, leading to larger errors in the estimated probabilities. Moreover, the criticality of each link differs, and its contribution to the variance in the importance weights is also variable. The non-linear cumulative effect of criticality leads to a disproportionate growth in the variance of the importance weights for failure scenarios with the increase in $\eta$. Therefore, adjusting the failure probabilities of critical links determined by $\eta$ rather than all links is essential for achieving more accurate probability estimates. In practice, to achieve more accurate probability estimates for extreme failure scenarios, it is advisable to use a relatively lower $\eta$. In our case study networks, $\eta$ taking the value of 0.1 or 0.15 is effective for achieving this accuracy. If it is required to further mitigate the impact of variance in importance weights on the accuracy of

**Table 5. Sampling results of GAE-IS in Chicago-Sketch road network**

| Network | Presampling time (hour) | | Sample size | $\theta_e$ | $\widehat{p}$ | VPE | $R$ |
|---|---|---|---|---|---|---|---|
| | Sub-network | Large-scale network | | | | | |
| CS | 15.48 | 493.37 | $1 \times 10^4$ | 16.68 | $8.64 \times 10^{-4}$ | $4.60 \times 10^{-4}$ | 2 |
| | | | $1 \times 10^4$ | 16.92 | $9.55 \times 10^{-6}$ | $2.37 \times 10^{-7}$ | 40 |

probability estimates, link criticality can be modified by employing scaling factors and constraining value ranges to reduce the fluctuations in the criticality of links. On the other hand, if the goal is to capture more extreme failure scenarios or achieve a smaller VPE, it may be beneficial to increase $\eta$, albeit with the understanding that this might introduce larger errors in probability estimation.

### Application of GAE-IS in the context of heterogeneous link failure probabilities

To examine the effectiveness of the proposed GAE-IS methodology, it was applied to the Chicago road network, specifically focusing on the failure probabilities of road segments due to seismic events. Since minor damage generally does not significantly affect road capacity,[54] this case study focuses on major damage to road segments. The failure probabilities of major damage to road segments were calculated based on the geographical seismic risk and the fragility curve of embankments. First, according to the mosaic-based Vs30 raster data from the Vs30 Map Viewer,[55] the Vs30 category of the site for each road segment was determined (Figure 6B). By integrating this category with the seismic hazard curve[56] (Figure 6E), the annual frequency curve of earthquakes exceeding a specified intensity for each road segment was derived. Subsequently, the seismic intensities were discretized, and based on the fragility curve[57] (Figure 6D), the number of major damage events per unit length of road segment within each intensity range was obtained. Leveraging these data, and assuming that earthquake occurrences follow a Poisson distribution, we calculated the conditional probability of failure for each road segment (Figure 6C). Detailed calculation methods can be found in Appendix C in the supplemental methods. Although we presented the risk of damage to only the CS network under seismic hazards, the estimation method for link failure probabilities can also be applied to other case networks. Ultimately, the failure probabilities of the road segments in the CS network were heterogeneous. However, in the CS network, the workflow of GAE-IS remained the same as in a network with homogeneous link failure probabilities, except that the structural failure probability, $\tau$, was replaced by heterogeneous link failure probabilities.

The results demonstrate that GAE-IS significantly outperforms crude Monte Carlo in sampling extreme failure scenarios with the same sample size (Figure 7). GAE-IS improved the sampling efficiency for extreme failure scenarios on the CS network by 2–40 times compared to crude Monte Carlo (as shown in Table 5). In addition, performing presampling on the sub-network took only 1/32 of the computational time required for presampling on the original network. These findings confirm that GAE-IS is both feasible and effective even in scenarios involving spatially heterogeneous link failure probabilities.

The GAE-IS methodology effectively decouples component criticality from vulnerability, allowing for straightforward updates to the ISD function in response to changes in seismic risk. Given a different seismic hazardous event, this process merely requires the integration of new link failure probabilities alongside their associated criticality.

While our primary focus has been on earthquakes, the framework of GAE-IS is versatile and can be applied to other disaster scenarios as well. Similar to updates necessitated by changes in seismic risk, adapting to different types of disasters does not require redoing presampling either. Instead, it suffices to replace the vulnerability distribution accordingly.

As a result, GAE-IS significantly reduces the cost of importance sampling for different disruptive events on the same network, making it a valuable tool for risk assessment and management.

### Features affecting link criticality in networks

The average results of feature perturbation for all links are shown in Figure 8. The results demonstrate that the remaining capacity of nodes is the most critical feature across all road networks, followed by the flow passing through the node, which ranks as the second most important feature. This pattern is consistent across different road networks, indicating that both the node capacity and the assigned traffic flow passing through the node are essential factors in identifying critical links for network functionality. Although the quantitative impact of these key factors on the estimated link likelihoods varies across different networks, this observation reinforces confidence in the potential to enhance the transferability of the GAE-IS method across various networks.

## DISCUSSION

This study proposes GAE-IS to estimate the probabilities of extreme failure scenarios in large-scale infrastructure networks, focusing on transportation systems to build up this methodology and investigate its performance. Central to our methodology is the development of the criticality assessor—a graph learning model that integrates topological centrality metrics with traffic flow attributes of network components to derive ISD functions. One of the key advantages of GAE-IS is its significantly lower computational cost for sampling extreme failure scenarios that lead to substantial performance degradation in network-structured infrastructure systems. This computational efficiency is largely attributed to the transferability of the criticality assessor. Additionally, the workflow of GAE-IS facilitates the decoupling of link criticality from failure risk. This separation allows for the integration of vulnerability distributions with link criticality distribution obtained through the criticality assessor. As a result, it facilitates a more streamlined approach to efficient acquisition of ISD

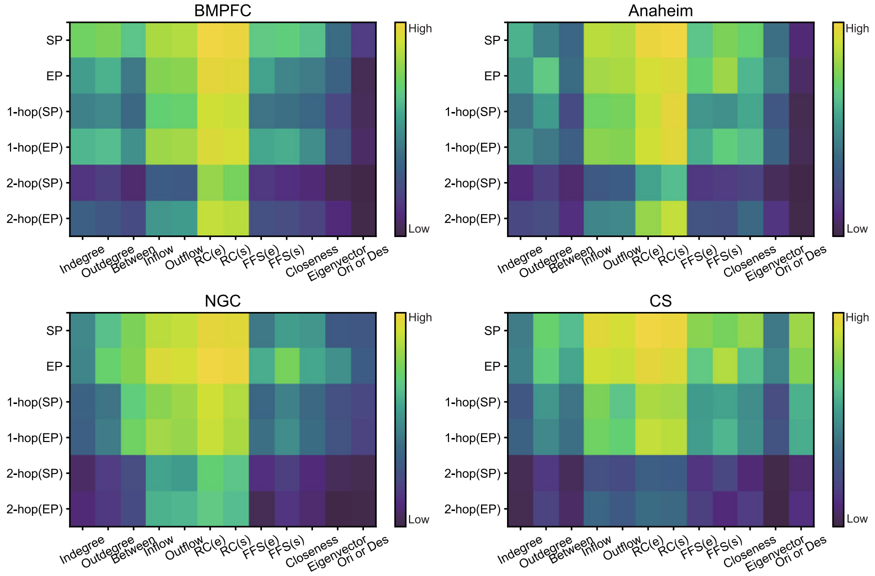


**Figure 8. Feature perturbation results for criticality assessors across different road networks, illustrating the contribution of different features to the criticality of road segments**

Warmer colors indicate higher importance, while cooler colors represent lower importance. "SP" refers to the starting point, "EP" denotes the ending point, "1-hop (SP)" represents the one-hop neighbors of the starting point, and "2-hop (SP)" refers to the two-hop neighbors of the starting point. Similarly, "1-hop (EP)" and "2-hop (EP)" represent the one-hop and two-hop neighbors of the ending point, respectively. "Between" refers to betweenness centrality, "RC (e/s)" refers to the remaining capacity of the node as an ending/starting point, and "FFS (e/s)" refers to the free-flow speed of the node as an ending/starting point.

functions, eliminating the need for extensive presampling and retraining of the model across various vulnerability distributions.

Extensive experiments demonstrate the effectiveness and efficiency of the proposed GAE-IS method for sampling extreme failure scenarios in road transportation networks. Experimental results on road networks with homogeneous link failure probabilities, such as those in Berlin, Anaheim, and the NGC, as well as on the Chicago road network with heterogeneous link failure probabilities under seismic risk, demonstrate that the GAE-IS method effectively captures more extreme failure scenarios. With a given sample size, it reduces the VPE and enhances sampling efficiency by one to two orders of magnitude, while also providing more accurate extreme failure probability estimates compared to crude Monte Carlo. Hence, the proposed GAE-IS method demonstrates significant promise for assessing the resilience of infrastructure networks from the perspective of extreme value statistics. Its design allows for straightforward extension to various types of infrastructure systems by adjusting the presampling of failure scenarios and the feature extraction for components in the objective network. For instance, future efforts can apply the proposed GAE-IS method to evaluate the extreme failure probability under a specific type of hazard for power grids, water distribution systems, etc. This flexibility enhances the method's applicability across different types of networks and research contexts.

The current GAE-IS is primarily applicable to infrastructure systems that have reached a mature stage, where the network's functionality and structure are well developed and the demand remains stable. If the system is still in the planning, construction, or operational testing phase, leading to significant changes in the network's topology, or if shifts in factors such as population structure, economic development, or lifestyle alter the spatial travel distribution pattern dramatically, retraining the model becomes necessary. This is because the criticality of network links may change as a result. However, the feature perturbation experiments for the criticality assessors reveal a consistent pattern in how node features, including topological centrality metrics and traffic flow attributes, contribute to link criticality. This phenomenon motivates further exploration of the transferability of GAE-IS across different networks with distinct topological structures and demand distribution patterns, such as networks that are undergoing development, as well as networks in different cities. Addressing this challenge and developing methods to build further transferability represent important directions for future research.

In conclusion, GAE-IS proves to be an effective and efficient tool for evaluating infrastructure system resilience. It is particularly applicable for systems that require substantial computational effort for their performance evaluation, especially in the context of analyzing extreme scenarios. The advantages of GAE-IS suggest promising applications in several key areas, including the design and operation of resilient infrastructure systems, the development of resilient cities, and the advancement of sustainable communities. Future research could explore further optimizations of the GAE-IS method to enhance its performance in diverse scenarios and expand its application across various infrastructure systems, such as water distribution networks, power grids, and communication systems. One promising direction involves refining the selection criteria of the sub-network for training the criticality assessor. Currently, approximately one-fourth to one-third of the original networks are randomly selected to serve as training networks for criticality assessors. We aim to explore a more systematic approach for sub-network selection, such as investigating the structural and functional similarities between sub-networks and the overall network, which could provide a theoretical basis for selecting training networks. By choosing more representative sub-networks that better capture the characteristics of the entire network, the performance of the GAE-IS method can be further enhanced.

## RESOURCE AVAILABILITY

### Lead contact

Requests for further information and resources should be directed to and will be fulfilled by the lead contact, Tingting Zhao (ttzhao@bjtu.edu.cn).

CellPress
OPEN ACCESS

**Materials availability**

This study did not generate new materials.

**Data and code availability**

The data for transportation networks can be accessed from the open-source transportation network dataset: https://github.com/bstabler/TransportationNetworks. The mosaic-based Vs30 raster data were obtained from the Vs30 Map Viewer,[55] while the seismic hazard curves were sourced from the USGS Earthquake Hazard Toolbox.[56] The code for GAE-IS is available at figshare.[58]

## ACKNOWLEDGMENTS

The authors disclose support for the research of this work from the National Natural Science Foundation of China (72288101, 72201028, 72242102, and 72091513) and the USA National Science Foundation (2047488).

## AUTHOR CONTRIBUTIONS

Conceptualization, T.Z., Z.G., and J.G.; data curation, M.G., T.Z., J.G., and X.M.; formal analysis, T.Z. and M.G.; funding acquisition, Z.G. and T.Z.; investigation, T.Z. and M.G.; methodology, T.Z., Z.G., and M.G.; project administration, resources, and supervision, Z.G. and T.Z.; visualization, T.Z. and M.G.; writing – original draft, T.Z. and M.G.; writing – review & editing, Z.G. and J.G.

## DECLARATION OF INTERESTS

The authors declare no competing interests.

## SUPPLEMENTAL INFORMATION

Supplemental information can be found online at https://doi.org/10.1016/j.patter.2025.101209.

Received: November 10, 2024
Revised: January 6, 2025
Accepted: February 27, 2025
Published: March 14, 2025

## REFERENCES

1. Montoya-Rincon, J.P., Mejia-Manrique, S.A., Azad, S., Ghandehari, M., Harmsen, E.W., Khanbilvardi, R., and Gonzalez-Cruz, J.E. (2023). A socio-technical approach for the assessment of critical infrastructure system vulnerability in extreme weather events. Nat. Energy *8*, 1002–1012.
2. Zheng, D., Tong, D., Davis, S.J., Qin, Y., Liu, Y., Xu, R., Yang, J., Yan, X., Geng, G., Che, H., and Zhang, Q. (2024). Climate change impacts on the extreme power shortage events of wind-solar supply systems worldwide during 1980-2022. Nat. Commun. *15*, 5225.
3. Jufri, F.H., Widiputra, V., and Jung, J. (2019). State-of-the-art review on power grid resilience to extreme weather events: Definitions, frameworks, quantitative assessment methodologies, and enhancement strategies. Appl. Energy *239*, 1049–1065.
4. Zhou, Y., Liu, K., and Wang, M. (2023). River flood risk assessment for the Chinese road network. Transport. Res. Transport Environ. *121*, 103818.
5. Tiedmann, H.R., Faust, K.M., and Sela, L. (2024). Looking beyond individual failures: A system-wide assessment of water infrastructure resilience to extreme events. Reliab. Eng. Syst. Saf. *244*, 109910.
6. Guddanti, B., Choi, J., Illindala, M.S., and Roychowdhury, R. (2022). Effect of endogenous failure events on the survivability of turboelectric distributed propulsion system. IEEE Trans. Ind. Appl. *58*, 224–232.
7. Shen, Y., Ren, G., and Ran, B. (2022). Analysis of cascading failure induced by load fluctuation and robust station capacity assignment for metros. Transportmetrica A: Transp. Sci. *18*, 1401–1419.
8. Hosseinalipour, S., Mao, J., Eun, D.Y., and Dai, H. (2020). Prevention and mitigation of catastrophic failures in demand-supply interdependent networks. IEEE Trans. Netw. Sci. Eng. *7*, 1710–1723.
9. Xu, Z., and Chopra, S.S. (2023). Interconnectedness enhances network resilience of multimodal public transportation systems for Safe-to-Fail urban mobility. Nat. Commun. *14*, 4291.
10. Poudyal, A., Poudel, S., and Dubey, A. (2023). Risk-based active distribution system planning for resilience against extreme weather events. IEEE Trans. Sustain. Energy *14*, 1178–1192.
11. Johnsson, I., and Balstrøm, T. (2021). A GIS-based screening method to identify climate change-related threats on road networks: A case study from Sweden. Clim. Risk Manag. *33*, 100344.
12. Stürmer, J., Plietzsch, A., Vogt, T., Hellmann, F., Kurths, J., Otto, C., Frieler, K., and Anvari, M. (2024). Increasing the resilience of the Texas power grid against extreme storms by hardening critical lines. Nat. Energy *9*, 526–535.
13. Ghosh, P., and De, M. (2024). Resilience-oriented planning for active distribution systems: A probabilistic approach considering regional weather profiles. Int. J. Electr. Power Energy Syst. *158*, 109976.
14. Pickering, E., Guth, S., Karniadakis, G.E., and Sapsis, T.P. (2022). Discovering and forecasting extreme events via active learning in neural operators. Nat. Comput. Sci. *2*, 823–833.
15. Owen, A. B. (2013). Monte Carlo theory, methods and examples. https://artowen.su.domains/mc/.
16. James, F. (1980). Monte Carlo theory and practice. Rep. Prog. Phys. *43*, 1145–1189.
17. Gong, X., Feng, S., and Pan, Y. (2023). An adaptive multi-fidelity sampling framework for safety analysis of connected and automated vehicles. IEEE Trans. Intell. Transport. Syst. *24*, 14393–14405.
18. Sun, J., Zhou, H., Xi, H., Zhang, H., and Tian, Y. (2022). Adaptive design of experiments for safety evaluation of automated vehicles. IEEE Trans. Intell. Transport. Syst. *23*, 14497–14508.
19. Song, J., Wei, P., Valdebenito, M., and Beer, M. (2021). Active learning line sampling for rare event analysis. Mech. Syst. Signal Process. *147*, 107113.
20. Xiang, Z., Chen, J., Bao, Y., and Li, H. (2020). An active learning method combining deep neural network and weighted sampling for structural reliability analysis. Mech. Syst. Signal Process. *140*, 106684.
21. Maddouri, O., Qian, X., Alexander, F.J., Dougherty, E.R., and Yoon, B.-J. (2022). Robust importance sampling for error estimation in the context of optimal Bayesian transfer learning. Patterns *3*, 100428.
22. Tokdar, S.T., and Kass, R.E. (2010). Importance sampling: a review. WIREs Comput. Stat. *2*, 54–60.
23. Fuh, C.D., and Wang, C.J. (2024). Efficient exponential tilting with applications. Stat. Comput. *34*, 65.
24. Cui, H., Tan, K.S., and Yang, F. (2024). Portfolio credit risk with Archimedean copulas: asymptotic analysis and efficient simulation. Ann. Oper. Res. *332*, 55–84.
25. Feng, S., Sun, H., Yan, X., Zhu, H., Zou, Z., Shen, S., and Liu, H.X. (2023). Dense reinforcement learning for safety validation of autonomous vehicles. Nature *615*, 620–627.
26. Zhao, D., Huang, X., Peng, H., Lam, H., and LeBlanc, D.J. (2018). Accelerated evaluation of automated vehicles in car-following maneuvers. IEEE Trans. Intell. Transport. Syst. *19*, 733–744.
27. Wang, Y. (2018). An adaptive importance sampling method for spinning reserve risk evaluation of generating systems incorporating virtual power plants. IEEE Trans. Power Syst. *33*, 5082–5091.
28. Wang, C., Xie, H., Bie, Z., Li, G., and Yan, C. (2021). Fast supply reliability evaluation of integrated power-gas system based on stochastic capacity network model and importance sampling. Reliab. Eng. Syst. Saf. *208*, 107452.
29. Gong, C., and Zhou, W. (2018). Importance sampling-based system reliability analysis of corroding pipelines considering multiple failure modes. Reliab. Eng. Syst. Saf. *169*, 199–208.

30. Zhang, Y., Song, K., Liu, D., Xiong, C., and Chou, S. (2023). A multi-mode failure boundary exploration and exploitation framework using adaptive kriging model for system reliability assessment. Probab. Eng. Mech. *73*, 103473.
31. Geyer, S., Papaioannou, I., and Straub, D. (2019). Cross entropy-based importance sampling using Gaussian densities revisited. Struct. Saf. *76*, 15–27.
32. Mehni, M.B., and Mehni, M.B. (2023). Reliability analysis with cross-entropy based adaptive Markov chain importance sampling and control variates. Reliab. Eng. Syst. Saf. *231*, 109014.
33. Zhang, J., Xiao, M., Gao, L., and Chu, S. (2019). A combined projection-outline-based active learning Kriging and adaptive importance sampling method for hybrid reliability analysis with small failure probabilities. Comput. Methods Appl. Mech. Eng. *344*, 13–33.
34. Bernard, D., and François, P. (2021). Safe adaptive importance sampling: A mixture approach. Ann. Stat. *49*, 885–917.
35. Papaioannou, I., Geyer, S., and Straub, D. (2019). Improved cross entropy-based importance sampling with a flexible mixture model. Reliab. Eng. Syst. Saf. *191*, 106564.
36. Au, S.K., and Beck, J.L. (2003). Important sampling in high dimensions. Struct. Saf. *25*, 139–163.
37. Tabandeh, A., Jia, G., and Gardoni, P. (2022). A review and assessment of importance sampling methods for reliability analysis. Struct. Saf. *97*, 102216.
38. Ottenburger, S.S., Cox, R., Chowdhury, B.H., Trybushnyi, D., Omar, E.A., Kaloti, S.A., Ufer, U., Poganietz, W.-R., Liu, W., Deines, E., et al. (2024). Sustainable urban transformations based on integrated microgrid designs. Nat. Sustain. *7*, 1067–1079.
39. Niu, C., Jian, S., Nair, D., and Dixit, V. (2023). Pre-disaster resilient road network investment strategy with uncertainty quantification. IEEE Trans. Intell. Transport. Syst. *24*, 6849–6864.
40. Coles, S. (2001). Classical Extreme Value Theory and Models. In An Introduction to Statistical Modeling of Extreme Values (Springer London), pp. 45–73.
41. Mohamad, M.A., and Sapsis, T.P. (2018). Sequential sampling strategy for extreme event statistics in nonlinear dynamical systems. Proc. Natl. Acad. Sci. USA *115*, 11138–11143.
42. Transportation Networks for Research Core Team (2016). Transportation networks for research. GitHub. https://github.com/bstabler/TransportationNetworks.
43. Wollenstein-Betech, S., Paschalidis, I.C., and Cassandras, C.G. (2022). Optimizing lane reversals in transportation networks to reduce traffic congestion: A global optimization approach. Transport. Res. C Emerg. Technol. *143*, 103840.
44. Xie, J., and Nie, Y.M. (2019). A new algorithm for achieving proportionality in user equilibrium traffic assignment. Transp. Sci. *53*, 566–584.
45. Xie, J., and Xie, C. (2015). Origin-based algorithms for traffic assignment: Algorithmic structure, complexity analysis, and convergence performance. Transp. Res. Rec. *2498*, 46–55.
46. Colon, C., Hallegatte, S., and Rozenberg, J. (2020). Criticality analysis of a country's transport network via an agent-based supply chain model. Nat. Sustain. *4*, 209–215.
47. Liu, Y., Song, A., Shan, X., Xue, Y., and Jin, J. (2022). Identifying critical nodes in power networks: A group-driven framework. Expert Syst. Appl. *196*, 116557.
48. Kipf, T.N., and Welling, M. (2016). Variational graph auto-encoders. Preprint at arXiv. https://doi.org/10.48550/arXiv.1611.07308.
49. Zhou, C., Liu, Y., Liu, X., Liu, Z., and Gao, J. (2017). Scalable graph embedding for asymmetric proximity. In Proc. Thirty-First AAAI Conference on Artificial Intelligence, pp. 2942–2948.
50. Salha, G., Limnios, S., Hennequin, R., Tran, V.-A., and Vazirgiannis, M. (2019). Gravity-inspired graph autoencoders for directed link prediction. In Proc. 28th ACM International Conference on Information and Knowledge Management, pp. 589–598.
51. Ou, M., Cui, P., Pei, J., Zhang, Z., and Zhu, W. (2016). Asymmetric transitivity preserving graph embedding. In Proc. 22nd ACM SIGKDD International Conference on Knowledge Discovery and Data Mining, pp. 1105–1114.
52. Vu, M., and Thai, M.T. (2020). PGM-Explainer: Probabilistic graphical model explanations for graph neural networks. Preprint at arXiv. https://doi.org/10.48550/arXiv.2010.05788.
53. Daganzo, C.F. (1997). Traffic flow theory. In Fundamentals of Transportation and Traffic Operations (Emerald Group Publishing Limited)), pp. 66–160.
54. Wang, H., Xiao, J., Li, S., and Zhai, C. (2024). Resilience assessment and optimization method of city road network in the post-earthquake emergency period. Earthq. Eng. Eng. Vib. *23*, 765–779.
55. Vs30 Map Viewer. https://usgs.maps.arcgis.com/apps/webappviewer/index.html?id=8ac19bc334f747e486550f32837578e1.
56. Earthquake Hazard Toolbox. https://earthquake.usgs.gov/nshmp/.
57. Maruyama, Y., Yamazaki, F., Mizuno, K., Tsuchiya, Y., and Yogai, H. (2010). Fragility curves for expressway embankments based on damage datasets after recent earthquakes in Japan. Soil Dynam. Earthq. Eng. *30*, 1158–1167.
58. Guo, M., Zhao, T., Meng, X., and Gao, Z. (2025). Code for GAE-IS. figshare. https://doi.org/10.6084/m9.figshare.27643302.v1.

Patterns, Volume *6*

# Supplemental information

# Quantifying extreme failure scenarios in transportation systems with graph learning

**Mingxue Guo, Tingting Zhao, Jianxi Gao, Xin Meng, and Ziyou Gao**

# Supplemental methods

### Appendix A: The formulation of User Equilibrium

This study takes the Average Travel Time (ATT) of the transportation network as the performance indicator. To calculate the ATT, we utilize User Equilibrium[1] to assign traffic demand in the network (Equations S1–S4). User Equilibrium (UE) assumes that road users have complete knowledge of the traffic conditions in the network and strive to choose paths with minimum travel cost. When the network reaches the state of user equilibrium, the travel times of all used paths for each origin-destination pair are equalized and minimized, and no user can unilaterally reduce their travel time by changing their route. The computation method of UE exemplifies the self-adaptability of travelers to congestion in the road network.

$$\min Z_{UE}(x) = \sum_i \int_0^{x_{l_i}} t_{l_i}(x)dx \qquad \text{(Equation S1)}$$

$$\text{s.t. } \sum_{k\in K^\omega} f_k^\omega = u^\omega, \ \ \forall\omega \in W \qquad \text{(Equation S2)}$$

$$x_{l_i} = \sum_\omega \sum_{k\epsilon K^\omega} \varpi_{l_ik}^\omega f_k^\omega \qquad \text{(Equation S3)}$$

$$f_k^\omega \geq 0, \forall k \in K^\omega, \omega \in W \qquad \text{(Equation S4)}$$

where $x_{l_i}$ represents the volume on road segment $l_i$; $t_{l_i}(x)$ is the cost function for road segment $l_i$, which in this case is the BPR function; $f_k^\omega$ denotes the flow on path $k$ for the origin-destination (OD) pair $\omega$; $u^\omega$ represents the total traffic demand for the OD pair $\omega$; $W$ denotes the set of all OD pairs, and $K^\omega$ represents the set of feasible paths for OD pair $\omega$; $\varpi_{l_ik}^\omega$ is a binary variable that takes the value 1 if path $k$ for OD pair $\omega$ passes through road segment $l_i$, and 0 otherwise. Equation S2 guarantees that the sum of path flows between an origin-destination (OD) pair equals the total traffic demand for that OD pair. Equation S3 denotes the composition relationship between a link flow and path flows going through this link. Equation S4 ensures that all path flows remain non-negative.

### Appendix B: Supplemental description of the GAE-IS workflow

If each Link $l_i$ in the target network has an expected structural failure probability denoted as $\tau_{l_i}(0 < \tau_{l_i} \leq 1)$, we employ the following approach for importance sampling.

(1) Assuming that each link in the training network has a homogeneous hypothetical failure probability $\epsilon$ $(0 < \epsilon < 1)$, the crude Monte Carlo is employed to randomly sample $N_t$ network failure scenarios with a link failure probability of $\epsilon$ as the original sample set.

(2) A predetermined percentage (the $\rho$ percentage) of original samples that demonstrate substantial degradation in network performance are selected as risk scenarios. The corresponding risk scenario threshold for Average Travel Time (ATT) is denoted as $\theta_r$.

(3) The weighted frequency of link occurrence within the failed link set in risk scenarios and the likelihood of links are computed as follows.

$$F_{l_i} = \sum_{k=1}^{\rho N_t} \frac{\epsilon N_a}{N_{fk}} \delta_{l_ik} \qquad \text{(Equation S5)}$$

$$h_{l_i} = \frac{F_{l_i}}{E(F)} = \frac{F_{l_i}}{\rho N_t \epsilon} \quad \text{(Equation S6)}$$

where $F_{l_i}$ represents the weighted frequency of Link $l_i$ occurring within the failed link set in risk scenarios; $N_{fk}$ represents the number of failed links in risk scenario $k$; $\epsilon$ is the hypothetical failure probability of links in the training network, $N_a$ represents the number of links in the training network, and $\epsilon N_a$ represents the expected number of failed links in a given scenario; $\delta_{l_i k}$ is a binary variable, if Link $l_i$ belongs to the failed link set in risk scenario $k$, $\delta_{l_i k}$ takes the value of 1; otherwise, it is 0; $h_{l_i}$ ($h_{l_i} > 0$) represents the likelihood of Link $l_i$ appearing in the failed link set in risk scenarios; $E(F)$ represents the expected number of occurrences of a link within the failed link set in risk scenarios.

(4) Using $h_{l_i}$ to adjust the failure probability of links, where each link now has a failure probability of $\min(h_{l_i}\epsilon, 1)$. Sampling $N_t$ network failure scenarios based on $\min(h_{l_i}\epsilon, 1)$ to create a new sample set.

(5) Select top $\rho$ of the new sample set that demonstrate substantial degradation in network performance as risk scenarios. If the corresponding risk scenario threshold $\theta_r'$ of the new sample set exceeds the extreme failure scenario threshold $\theta_e$, execute Step (6); otherwise, return to Step (3).

(6) Train Criticality Assessor with $h_{l_i}$ as the link label. A trained Criticality Assessor can be transferred to the target network and outputs the estimated $\hat{h}_{l_i}$ for the target network.

(7) The $\hat{h}_{l_i}$ of all links is sorted in descending order. The top $\eta$ proportion of links are critical links, and the bottom $\eta$ proportion of links are non-critical links. Then, $\hat{h}_{l_i}$ will be modified as follows:

$$c_{l_i} = \begin{cases} \frac{2\eta N \hat{h}_{l_i}}{\sum_{l_i \in B} \hat{h}_{l_i}} & l_i \in B \\ 1 & l_i \notin B \end{cases} \quad \text{(Equation S7)}$$

where $c_{l_i}$ is the modified $\hat{h}_{l_i}$, $N$ is the number of links in the target network, $B$ is the set of critical and non-critical links.

(8) Based on $c_{l_i}$ and $\tau_{l_i}$, perform importance sampling to generate failure scenarios and estimate the probability of extreme failure scenarios in the target network.

## Appendix C: The seismic failure probability of road segments in Chicago road network

The average shear wave velocity for the top 30 meters (Vs30) is an important parameter in seismic engineering. It can be used to evaluate the amplification and attenuation characteristics of seismic waves propagating underground[2,3], which is crucial for seismic risk analysis. According to Vs30, the site can be divided into different categories, and determining the site category is a key step in analyzing site seismic effects. Therefore, Vs30 is widely applied as an important site parameter in various fields of seismic engineering, such as seismic risk assessment and seismic design of buildings[4]. In this study, the Vs30 data for Chicago is derived from the mosaic-based Vs30 raster data provided by the Vs30 Map Viewer[5] of the United States Geological Survey (USGS). The Vs30 category of the site for each road segment is determined

by the predominant Vs30 category among the raster cells traversed by the segment (Figure 6B in the main text).

USGS Earthquake Hazard Toolbox is the "web applications for querying and computing hazard from USGS national seismic hazard models" (https://earthquake.usgs.gov/nshmp/). It provides seismic hazard curves for different site categories within the United States. The seismic hazard curve for Chicago is retrieved as shown in Figure 6E in the main text. The horizontal axis represents the peak ground velocity (PGV in cm/s), indicating the maximum velocity of ground motion during an earthquake. It is a parameter that represents the intensity of earthquakes and can be used to assess the impact of earthquakes on buildings and structures. The vertical axis represents the annual frequency of earthquakes exceeding a specific PGV. Based on the Vs30 category of the site for each road segment, the corresponding seismic hazard curve can be obtained.

The seismic damage to urban roads is usually caused by ground displacement. Due to the lack of data on possible ground deformation in Chicago, we use the vulnerability curve of road embankments to measure seismic damage to roads. Maruyama et al.[6] fitted a vulnerability curve for embankments based on recorded data from actual earthquake disasters. This curve reflects the number of major damage incidents per unit length of the embankment at different PGVs (Figure 6D in the main text). Their study is also documented in the European funded research project "Systemic Seismic Vulnerability and Risk Analysis for Buildings, Lifeline Networks and Infrastructures Safety Gain" (SYNER-G)[7]. In seismology, earthquake occurrences are often regarded as a Poisson process, thus the failure probability $\zeta$ of a link can be expressed as the Equation S8[8]:

$$\zeta = 1 - e^{-\lambda\iota} \qquad \text{(Equation S8)}$$

where $\lambda$ represents the number of major damage incidents per unit length of the embankment, which is related to PGV; $\iota$ is the length of the link.

We compute failure probabilities for road segments in Chicago under the condition of earthquakes with PGV in the range of 4-525 cm/s. Based on the data points along the seismic hazard curve, PGV has been segmented into 12 intervals. The annual frequency of earthquakes with PGV in a specific interval can be calculated by taking the difference in annual frequency of exceedence between interval endpoints. The number of major damage incidents per unit length of the embankment in each PGV interval is represented by the $\lambda$ of the median PGV of each interval. Finally, the structural failure probability $\tau$ for a road segment under the condition of earthquakes with PGV in the range of 4-525 cm/s can be calculated using the Equation S9.

$$\tau = \frac{\sum_{i=1}^{12} \chi_i \zeta_i}{\sum_{i=1}^{12} \chi_i} \qquad \text{(Equation S9)}$$

where $\chi_i$ represents the annual frequency of earthquakes with PGV falling within the $i$-th interval; $\zeta_i$ represents the failure probability of a link with PGV falling within the $i$-th interval.

Moreover, the failure probability of all connector links from the zone centroid to the surrounding links is set to be 0.

**Appendix D: Training iteration, loss function and VPE of Criticality Assessor**

Figure S1 illustrates the variation trend of the loss function and the VPE for extreme failure scenarios versus number of epochs.

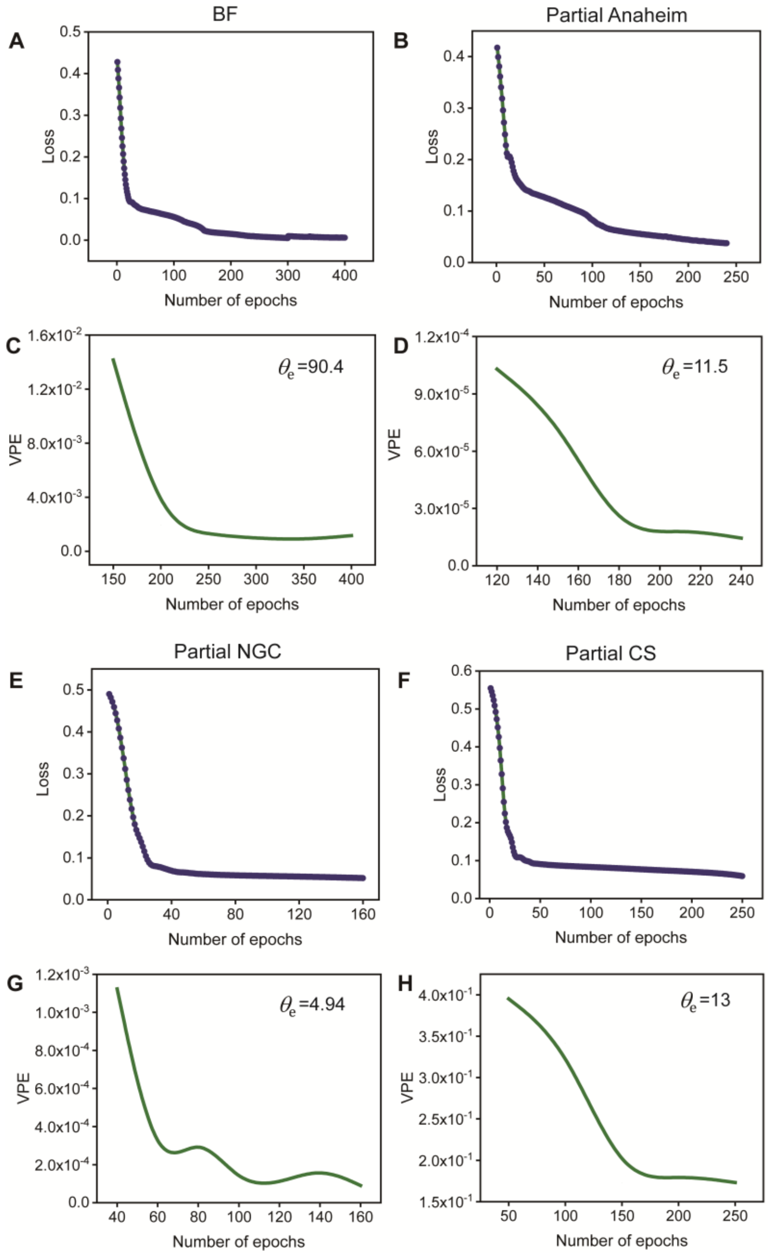


Figure S1: Values of loss function over epochs in the training of Criticality Assessor and the VPE for extreme failure scenarios with respect to each epoch.

(A) Loss function curve when training Criticality Assessor on the BF network.

(B) Loss function curve when training Criticality Assessor on the partial Anaheim network.

(C) VPE of BF network over epochs with the Criticality Assessor trained in (A).

(D) VPE of partial Anaheim network over epochs with the Criticality Assessor trained in (B).

(E) Loss function curve when training Criticality Assessor on partial NGC network.

(F) Loss function curve when training Criticality Assessor on partial CS network.

(G) VPE of partial NGC network over epochs with the Criticality Assessor trained in (E).

(H) VPE of partial CS network over epochs with the Criticality Assessor trained in (F).

**Supplemental references**


1. Wardrop, J. G (1952). Some theoretical aspects of road traffic research. In ICE Proceedings of Engineering Divisions. pp. 325-378.
2. Alvarado, P., Christiansen, R., Gregori, S. D., and Saez, M. (2020). Evidence of site amplification from ground motion of the last two large crustal earthquakes in central-western Argentina. Nat. Hazards *102*, 1011-1031.
3. Karimzadeh, S., Feizizadeh, B., and Matsuoka, M. (2017). From a GIS-based hybrid site condition map to an earthquake damage assessment in Iran: Methods and trends. Int. J. Disaster Risk Reduc. *22*, 23-36.
4. Abbasnejadfard, M., Bastami, M., Jafari, M. K., and Azadi, A. (2023). Spatial correlation models of VS30 values: A case study of the Tehran region. Eng. Geol. *325*, 107300.
5. Vs30 Map Viewer. https://usgs.maps.arcgis.com/apps/webappviewer/index.html?id=8ac19bc334f747e486550f32837578e1.
6. Maruyama, Y., Yamazaki, F., Mizuno, K., Tsuchiya, Y., and Yogai, H. (2010). Fragility curves for expressway embankments based on damage datasets after recent earthquakes in Japan. Soil Dyn. Earthq. Eng. *30*, 1158-1167.
7. Kaynia, Amir M., Iervolino, I., Taucer F., and Hancilar, U. (2013). Guidelines for deriving seismic fragility functions of elements at risk – Buildings, lifelines, transportation networks and critical facilities. Publications Office of the European Union. https://publications.jrc.ec.europa.eu/repository/bitstream/JRC80561/lbna25880enn.pdf.
8. Wisetjindawat, W., Kermanshah, A., Derrible, S., and Fujita, M. (2017). Stochastic Modeling of Road System Performance during Multihazard Events: Flash Floods and Earthquakes. J. Infrastruct. Syst. *23*, 04017031.
